# A multimodal *in situ* framework for spatial characterization applied on SARS-CoV-2 live-attenuated vaccines in the nasal mucosa

**DOI:** 10.1016/j.isci.2026.117730

**Published:** 2026-09-22

**Authors:** Tobias Britzke, Jacob Schön, Nico Joël Halwe, Lorenz Ulrich, Jana Kochmann, Björn Corleis, Robert Klopfleisch, Martin Beer, Donata Hoffmann, Angele Breithaupt

**Affiliations:** 1 Department of Experimental Animal Facilities and Biorisk Management, Friedrich-Loeffler-Institute, Greifswald-Insel Riems, Germany; 2 Institute of Diagnostic Virology, Friedrich-Loeffler-Institute, Greifswald-Insel Riems, Germany; 3 Institute of Immunology, Friedrich-Loeffler-Institute, Greifswald-Insel Riems, Germany; 4 Institute of Veterinary Pathology, Freie Universität Berlin, Berlin, Germany

**Keywords:** respiratory virus infection, SARS-CoV-2, live-attenuated vaccine, virus-host interactions, nasal mucosa, spatial analysis, histopathology, RNA *in situ* hybridization, immunohistochemistry, quantitative image analysis

## Abstract

Respiratory viruses frequently initiate infection in the nasal mucosa, where virus-host interactions shape disease progression and transmission. Yet this site remains undercharacterized due to anatomical and technical challenges. Spatially resolved technologies are limited by cost and complexity, leaving most studies reliant on bulk or descriptive approaches lacking spatial context. We present a framework for spatially resolved investigation of infections in the nasal cavity. Using wild-type SARS-CoV-2 and two live-attenuated vaccines (OTS-206, OTS-228) as model viruses in Syrian hamsters, we integrate standardized sampling, histopathology, immunohistochemistry, multiplex RNA *in situ* hybridization, and quantitative image analysis across spatial scales, including tissue, single-cell, and subcellular resolution. We identified distinct infection patterns and graduated tissue damage from wild-type to OTS-228. Despite limited infection, OTS-228 induces a robust tissue-preserving immune response. Subcellular analysis shows that both vaccines exhibit a primarily cytoplasmic distribution of host antiviral transcripts, whereas WT infection is characterized by predominantly nuclear RNA localization.

## Highlights

• Standardized nasal cavity analysis provides consistent histopathological readouts
• Modular framework integrates complementary approaches for spatial tissue evaluation
• Scalable and adaptable framework for different research questions and resources
• Expert pathology ensures accurate interpretation at tissue and (sub)cellular levels

## Graphical abstract

**Figure:**
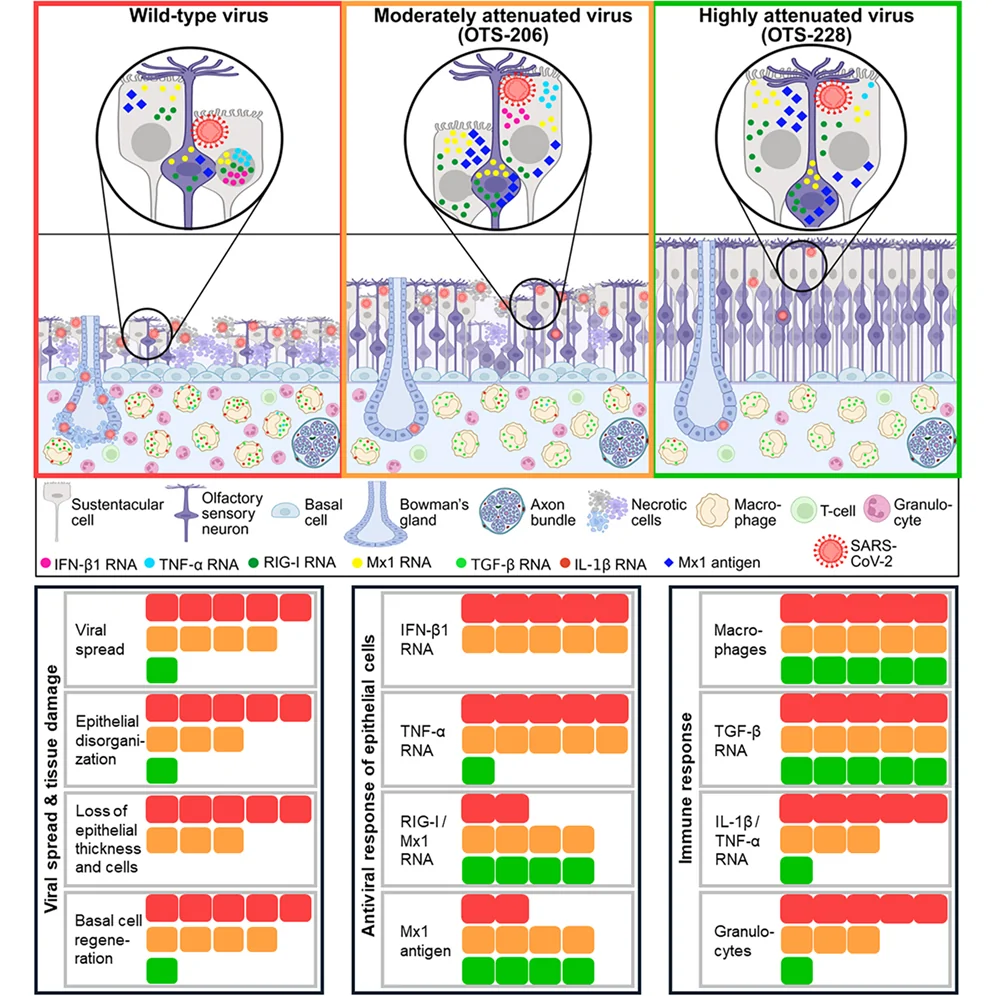

## Teaser

Immunology; Virology; Histopathology/Pathology

## Introduction

The nasal mucosa represents the initial replication site for many emerging respiratory viruses, serves as a key interface for pathogen-host interaction and a gateway to the lung, the brain, or systemic spread.^1^ Despite this central role, the nasal cavity remains comparatively undercharacterized relative to the lower respiratory tract in many experimental infection studies.^2^ This imbalance became particularly evident during the COVID-19 pandemic, when the Syrian hamster was widely used as a model for pathology, vaccination, and transmission,^3^ yet most studies focused predominantly on lung pathology and viral loads.^4^^,^^5^^,^^6^ This gap largely reflects the complex anatomy, functional architecture, and technical challenges in tissue processing and analysis, including its three-dimensional turbinate structure and susceptibility to artifacts.^7^^,^^8^^,^^9^^,^^10^ Reliable interpretation requires specific morphological expertise and standardized evaluation across defined anatomical levels.^2^^,^^11^^,^^12^ Also, the transient nature of infection complicates the selection of appropriate sampling time points.^13^ For studies on viral tropism, transmission, and local immunity, the spatial analysis of Syrian hamster nasal tissues is particularly relevant.

Recent advances in spatially resolved technologies enable high-resolution analysis of virus-host interactions,^14^^,^^15^ but remain technically demanding, costly, and difficult to apply to complex tissues and infection models under high biosafety conditions.^14^^,^^16^^,^^17^ Some studies provided insights into SARS-CoV-2-associated tissue responses, particularly in the lung and selected human tissues,^18^^,^^19^^,^^20^ but their impact on analysis, particularly in complex tissues and under BSL-3 or BSL-4 conditions, has remained limited despite their considerable potential.

Many studies of respiratory infections rely on descriptive histopathology, with some integrating immunohistochemistry (IHC) for viral antigens or immune cells, but mostly without standardized processing and predefined regions of interest (ROIs).^21^^,^^22^^,^^23^^,^^24^^,^^25^^,^^26^ Only a few studies applied standardized trimming strategies,^27^ or predefined compartments for analysis.^8^^,^^10^^,^^28^ The lack of standardized protocols for sampling and analysis can compromise reproducibility and interpretability.^29^ Further, multiplex antigen detection, RNA *in situ* hybridization (ISH), and digital image analysis have expanded the toolbox but are typically limited to virus detection, selected markers, or lung-focused studies.^21^^,^^22^^,^^26^^,^^30^ Host gene expression in the nasal mucosa is rarely analyzed *in situ* and is mostly studied in mouse models.^30^^,^^31^^,^^32^ Instead, transcriptional responses are typically assessed by bulk methods (qPCR or RNA-seq), which lack spatial resolution and cannot assign gene expression to defined structures or infection sites.^28^^,^^33^^,^^34^

Thus, a standardized framework that integrates structured tissue sampling and anatomically defined ROIs with qualitative, quantitative, and spatially resolved analyses of the nasal cavity is lacking. To address this gap, we established a broadly applicable methodological approach to achieve a new level of spatial resolution in the analysis of respiratory virus infections in this complex tissue. To demonstrate its applicability in a biological context, we used highly virulent wild-type (WT) SARS-CoV-2 and two live-attenuated vaccine (LAV) candidates, OTS-206 (moderately attenuated) and OTS-228 (highly attenuated), as model viruses in the Syrian hamster. Both LAVs were generated using the one-to-stop (OTS) genome recoding attenuation strategy and further harbor disruptive mutations in NSP1 and deletions of ORFs 6–8, while OTS-228 additionally lacks the polybasic furin cleavage site (FCS).^35^^,^^36^^,^^37^ In Syrian hamsters, OTS-206 was moderately attenuated but transmissible, whereas OTS-228 was highly attenuated and non-transmissible.^37^^,^^38^

By integrating anatomical mapping, histopathology, IHC, multiplex ISH combined with IHC, and quantitative digital image analysis, our framework is designed in a modular setup to characterize viral infection patterns, virus-induced lesions, host immune responses, and cell-type-specific interactions within the same anatomical context across multiple levels of resolution: from tissue to single-cell and subcellular readout. This modular design is intended to facilitate scalability, as analyses can be omitted, expanded, or adapted according to the scientific question, available resources, or initial findings. Cost-effective initial analyses allow hypothesis refinement and guide the targeted application of more costly, high-resolution methods to the same sample set when deeper mechanistic insights are required. Because the framework is based on general principles of tissue-based pathogenesis research, it is considered readily adaptable to other respiratory viruses when pathogen-specific markers and biological readouts are selected.

## Results

### A structured spatial analysis enables consistent *in vivo* analysis of respiratory virus infection in the nasal cavity

As outlined in Figure 1 and detailed in the Materials and Methods, we analyzed the nasal tissue at three standardized anatomical levels. To ensure consistency in histopathological and digital image analyses while maximizing tissue coverage, we predefined seven regions of interest (ROIs) (Figure 1A). To spatially correlate the parameters, serial consecutive sections were examined for each ROI in all animals (Figure 1B). Most techniques applied in this study are routinely available in research pathology laboratories, including hematoxylin and eosin staining (HE) and antigen detection by immunohistochemistry (IHC) or immunofluorescence, by single or double labeling. These approaches allow assessment of tissue damage and identification of immune and target cells by bright-field or fluorescence microscopy (Figure 1B). To extend beyond descriptive and semi-quantitative analyses, we performed quantitative whole-slide image analysis to measure infection, tissue damage, regeneration, and immune cell infiltration. In addition, multiplex RNA *in situ* hybridization (ISH) combined with antigen detection was used to analyze gene expression in defined cell populations and their microenvironment by confocal microscopy (Figure 1B). Spatially resolved analysis from anatomical or functional compartments to cellular and subcellular levels requires expertise in tissue composition and the interpretation of antigen and genome detection, as depicted in Figure 1C. This framework was applied to assess the expected graded viral attenuation and the effects of genetic modifications on the olfactory and respiratory mucosa of Syrian hamsters using the highly virulent wild-type (WT) SARS-CoV-2 virus and the moderately attenuated OTS-206 and highly attenuated OTS-228 vaccine viruses.

**Figure 1:**
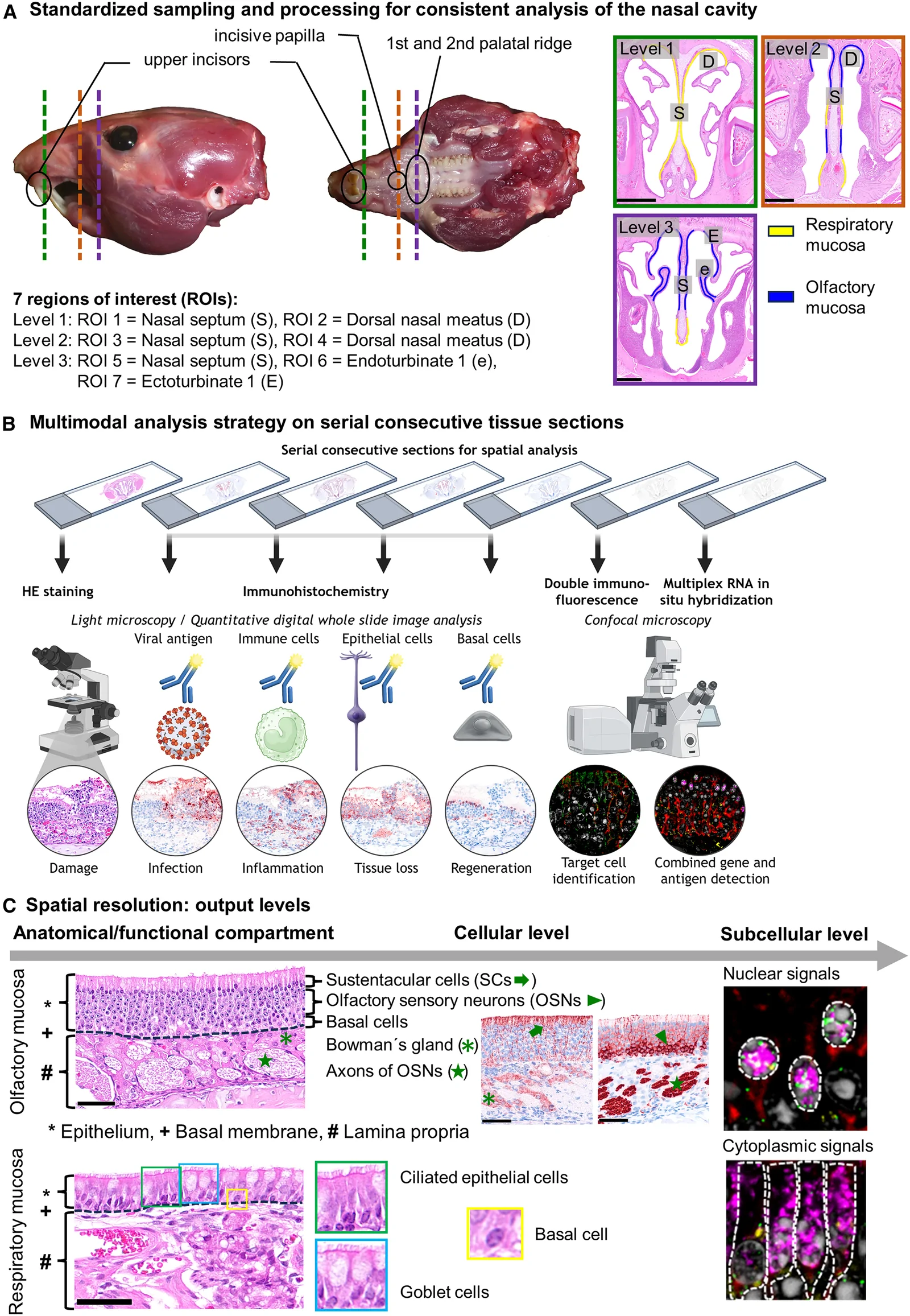
Multimodal framework to study respiratory infections in the nasal cavity A standardized sampling, processing and analysis strategy for spatial evaluation of the nasal mucosa in the Syrian hamster model. (A) Following 7-day formalin fixation and 21-day EDTA-based decalcification, three transverse levels were defined using anatomical landmarks: level 1 (green box), posterior to the upper incisors, lined by respiratory epithelium (in yellow); level 2 (brown box), at the incisive papilla, with equal proportions of respiratory epithelium and olfactory epithelium (in blue); and level 3 (purple box), between the first and second palatal ridge, lined mainly by olfactory epithelium. Seven regions of interest (ROIs) were defined: levels 1 and 2 = nasal septum ‘S’ and dorsal nasal meatus ‘D’; level 3 = nasal septum ‘S’, endoturbinate 1 ‘e’, and the dorso-medial surface of ectoturbinate 1 ‘E’ (scale bars, 1 mm, hematoxylin and eosin stain, HE). (B) Analysis strategy: serial consecutive sections from each level were used for multimodal analysis to assess tissue damage; immunohistochemistry for viral antigen, immune, epithelial, and basal cells with digital image analysis; double immunofluorescence for target cell identification; and multiplex RNA *in situ* hybridization combined with antigen detection to analyze host gene expression in spatial context. Created in BioRender. Britzke, T. (2026) https://BioRender.com/b602vx0. (C) Spatial analysis covered anatomical and functional compartments, cellular and subcellular levels using light and confocal microscopy. The nasal mucosa comprises a pseudostratified epithelium and underlying lamina propria separated by a basal membrane. The olfactory epithelium contains sustentacular cells (SCs), olfactory sensory neurons (OSNs), and basal progenitor cells; OSN nuclei are arranged in layers beneath a single SC layer, with cytoplasmic processes of both extending from the basal membrane to the luminal surface. The lamina propria contains OSN axon bundles and Bowman’s glands. The respiratory epithelium comprises ciliated epithelial cells, mucus-secreting goblet cells, and basal progenitor cells. The basal membrane served as an infection- and lesion-independent reference for quantitative image analysis (scale bars, 50 μm).

In practice, accurate morphological assessment of the nasal cavity required considerable histopathological expertise and time, which may not be available in all research settings. Digital image analysis likewise depended on solid pathomorphological knowledge, as automated annotation, particularly in the complex and artifact-prone nasal cavity, was often unreliable, necessitating manual correction and expert validation of both annotations and subsequent quantifications.

### Quantitative and qualitative viral antigen analysis identifies potential shifts and changes in target tissues and cells

The experimental setup for intranasal inoculation with WT, OTS-206 or OTS-228 attenuated live vaccine is depicted in Figure S1A. Significant differences have been observed in clinical signs and viral genome loads, with the highest in WT, the lowest in OTS-228, and OTS-206 in between. Details on body weight, viral genome shedding, viral genome loads in tissues, and humoral immune response are given in the Note S1; Figure S1. Mock inoculated hamsters served as negative controls. Together with findings from previous *in vivo* and *in vitro* studies,^37^^,^^38^ this indicates differential infection dynamics between OTS-206 (with FCS) and OTS-228 (without FCS).

The deletion of the FCS in OTS-228 not only prevents virus transmission,^37^ but also likely influences the extent of nasal mucosal infection and may also alter tissue and cell tropism.^39^^,^^40^ To address this common question of extent and target cell tropism, we quantified the area of SARS-CoV nucleoprotein (SARS-NP) positive cells with software-based image analysis in the olfactory and respiratory mucosa within predefined areas. Seven ROIs were selected based on consistent anatomical landmarks and their known susceptibility to SARS-CoV-2 infection.^8^^,^^10^ Values were normalized to 100 μm of basal membrane, which served as an infection- and lesion-independent reference structure. To detect shifts in target cell tropism, we colocalized SARS-NP with cytokeratin 18 (CK18, for sustentacular cells, SCs) or neuron-specific class III β-tubulin (Tuj1, for olfactory sensory neurons, OSNs) in the olfactory epithelium.

Detailed results are provided in Figure 2. Briefly, at 2 days post-inoculation (dpi), WT and OTS-206 yielded significantly larger antigen labeled areas compared to OTS-228 in the olfactory epithelium (Figure 2A). In the respiratory epithelium, SARS-NP labeling revealed large areas of positive epithelial cells for OTS-206, without statistical significance compared to WT but significantly higher than for OTS-228 (Figure 2B). Determination of the infection pattern revealed WT and OTS-206 showing a multifocal to coalescent, or diffuse SARS-NP distribution (Figures 2C and 2D), but for OTS-228, only single cells or a few sharply demarcated clusters were observed (Figure 2E). Target cells comprised mainly ciliated epithelial cells in the respiratory epithelium for all viruses. In the olfactory mucosa, WT antigen labeling in Bowman’s glands was significantly more widespread compared to both OTS variants (Figures 2F and 2G), and only WT antigen was found in the vomeronasal organ (Figure 2H). Traces of cytoplasmic antigen in macrophages within the propria and the nasal-associated lymphoid tissue were found across all animals at 2 dpi (Figure 2I). Immunofluorescence double labeling of the olfactory epithelium revealed sustentacular cells (SCs) to be the main target of all variants (Figure 2J). Only a few SARS-CoV-2 positive olfactory sensory neurons (OSNs) were identified following OTS-206 and OTS-228 inoculation (Figure 2K). Furthermore, no viral antigen was found in axon bundles of the propria in any hamster, which ultimately traverse to the olfactory bulb of the brain.

**Figure 2:**
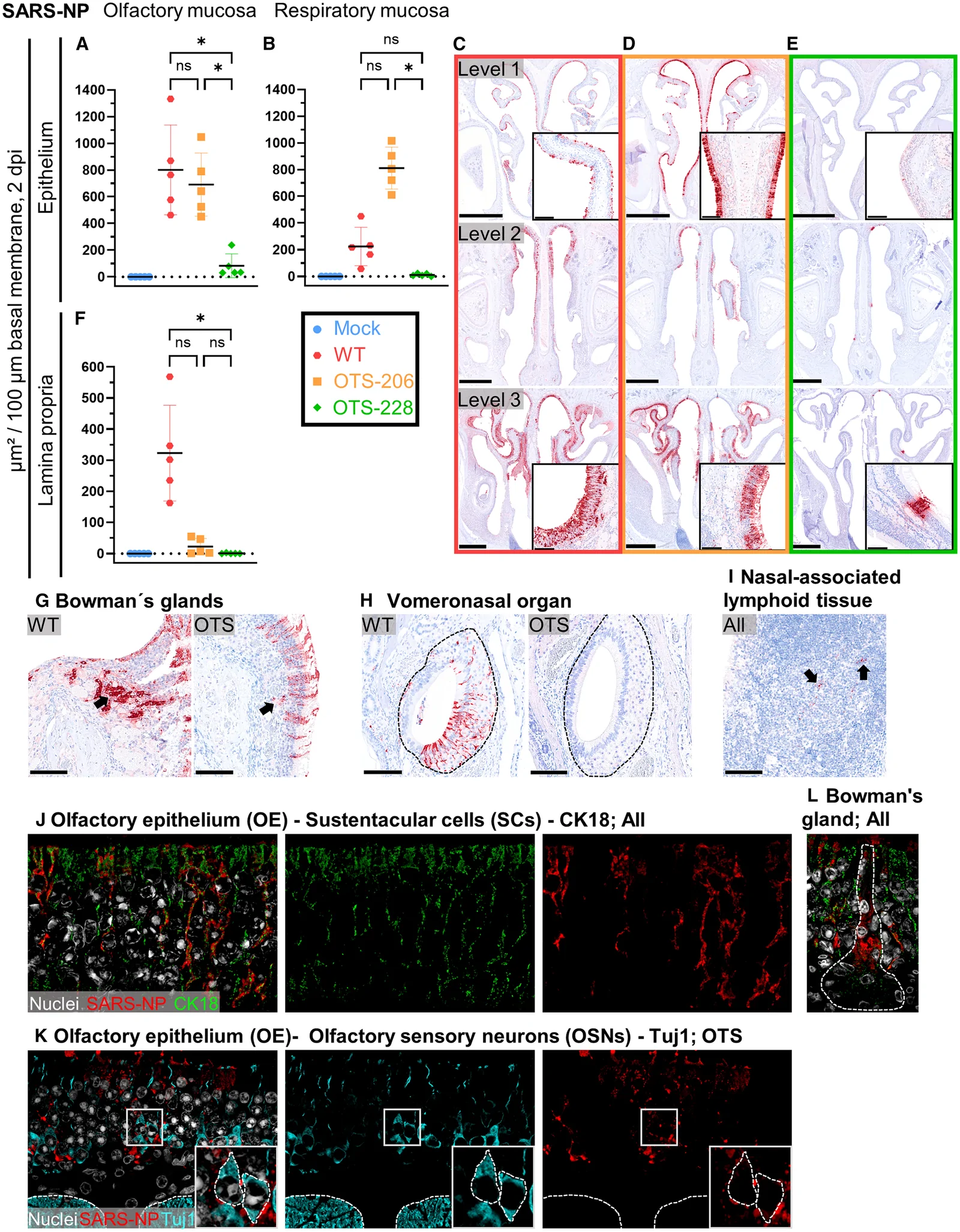
Viral protein distribution and target cell tropism in the nasal mucosa at 2 dpi (A) SARS-NP positive areas in the olfactory epithelium were comparable between WT and OTS-206 inoculated hamsters and significantly larger than in OTS-228. (B) In the respiratory epithelium, larger SARS-NP positive areas were observed after OTS-206 than after WT inoculation. (C–E) Representative sections illustrate multifocal to diffuse SARS-NP labeling in epithelial cells of the olfactory epithelium (levels 2–3) and respiratory epithelium (levels 1–2; predominantly ciliated epithelial cells) following (C) WT and (D) OTS-206 inoculation. In contrast, (E) OTS-228 antigen was restricted to single cells or oligofocal clusters of few epithelial cells (overview, scale bars, 1 mm; detail, scale bars, 100 μm). (F) In the lamina propria of the olfactory mucosa, abundant viral antigen was detected after WT inoculation, whereas only minimal antigen was observed after OTS-206 and OTS-228. (G) WT led to prominent infection of numerous Bowman’s gland epithelial cells (black arrow) and only individual virus-positive glandular cells following OTS inoculation (scale bars, 100 μm). (H) The vomeronasal organ (black dotted contour) was affected in all WT-infected hamsters, but not in OTS-inoculated hamsters (scale bars, 100 μm). (I) SARS-NP antigen (black arrow) was detected in the cytoplasm of macrophages of the nasal-associated lymphoid tissue in all groups (scale bars, 100 μm). (J–L) Representative immunofluorescence images (63× magnification) of the olfactory epithelium identified (J) sustentacular cells (CK18, green) as the main target cell (SARS-NP, red) for all groups. (K) Few SARS-NP positive olfactory sensory neurons (Tuj1, cyan; magnified image, white dotted contour) were detected, while axon bundles in the lamina propria (overview image, white dotted contour) remained negative following OTS inoculation. (L) SARS-NP positive Bowman’s glands (CK18, green; white dotted contour). Kruskal-Wallis test followed by Dunn’s correction was performed (A, B, F). ∗*p* < 0.05, ns = not significant. Mean with standard deviation is shown. SARS-NP: SARS-CoV nucleocapsid protein; CK18: cytokeratin peptide 18; Tuj1: neuron-specific class III beta-tubulin. *n* = 5 hamsters per group; 2 dpi; nasal levels 1, 2, and 3, with a total of 7 regions of interest, were analyzed.

Overall, in line with PCR data on tissue samples at 2 dpi, WT and OTS-206 resulted in widespread and comparable SARS-NP positive areas in the nasal mucosa, but OTS-228 infection was restricted. Our readout complemented virology data with the distribution pattern indicating a limited cell-to-cell spread of OTS-228. Moreover, PCR data did not resolve that OTS tended to shift from the olfactory to the respiratory epithelium, while no evidence of altered main target cell tropism was detected.

### Multiple morphological parameters of infection-related tissue alterations in olfactory and respiratory mucosa

Next, we correlated the extent of infection with associated tissue damage and regeneration, to gain insights into potential functional consequences in the olfactory epithelium (Figure 3) and respiratory epithelium (Figure S2). To this end, we recorded nasal lesions and assessed four morphological parameters requiring varying levels of histological expertise, supported where appropriate, by digital image analysis: (1) epithelial disorganization (Figures 3A–3E and S2A–S2E) and (2) epithelial thickness (Figures 3F and S2F) of the olfactory epithelium and respiratory epithelium, as well as (3) loss of SCs (Figure 4A) or OSNs (Figure 4B), and (4) basal cell driven regeneration (Figure 4C) of the olfactory epithelium. Due to tissue complexity, disorganization and regeneration were assessed semi-quantitatively by manual evaluation, whereas epithelial thickness and the area of targeted cell populations per 100 μm basal membrane were quantified by digital image analysis within seven predefined ROIs at 2, 5 and 21 dpi. Mock-inoculated animals defined the physiological range.

**Figure 3:**
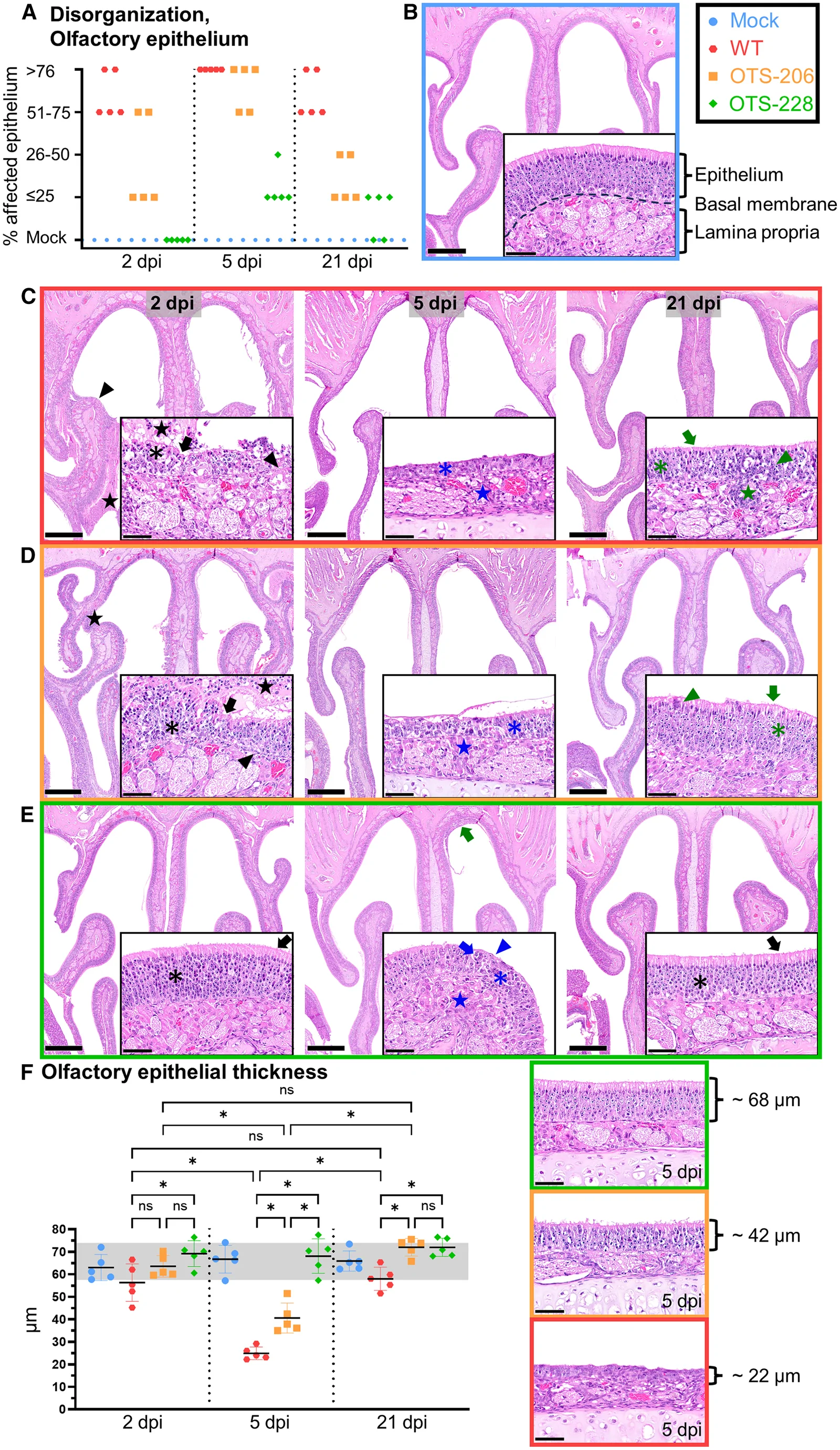
Histopathology of the olfactory mucosa at 2, 5 and 21 dpi (A) Disorganization of the olfactory epithelium, given in %, showing gradual tissue damage for WT, OTS-206, and OTS-228. (B–E) Representative hematoxylin and eosin-stained sections of level 3 (overview, scale bars, 500 μm; detail, scale bars, 50 μm) to illustrate lesions in the olfactory mucosa. (B) Mock-inoculated hamsters with well-structured olfactory mucosa. (C) WT: At 2 dpi, widespread disruption of olfactory epithelium architecture with epithelial necrosis (black asterisk), diffuse loss of the ciliary layer, and irregular luminal surface (black arrow), epithelial exfoliation, and exposure of the basal membrane (black arrowhead), luminal cellular debris, proteinaceous mucus, and degenerated granulocytes and macrophages (black star). At 5 dpi, severe loss of sustentacular cells (SCs) and olfactory sensory neurons (OSNs) with remaining multilayered basal cells (blue asterisk) and widespread necrosis of Bowman’s glands (blue star). At 21 dpi, substantial recovery of the epithelium (green asterisk) and ciliary layer (green arrow), with regeneration of Bowman’s glands (green star), while SCs and OSNs remained markedly disorganized (green arrowhead). (D) OTS-206: At 2 dpi, the basal membrane remained largely covered (black arrowhead), with epithelial necrosis and disorganization (black asterisk), loss of the ciliary layer, and irregular luminal surface (black arrow), and epithelial exfoliation into the nasal cavity (black star). At 5 dpi, severe but incomplete loss of SCs and OSNs (blue asterisk) with largely preserved Bowman’s glands (blue star). At 21 dpi, a largely restored olfactory epithelium (green asterisk) with recovered ciliary layer (green arrow) and only mild irregularities in SC and OSN organization (green arrowhead). (E) OTS-228: Preserved, continuous epithelial (black asterisk) and ciliary layers (black arrow) and intact Bowman’s glands (blue star), with only minimal alterations, including oligofocal single-cell necrosis (blue arrow), mild epithelial disruption by immune cell infiltration (blue asterisk), and focal ciliary loss (blue arrow) at 5 dpi. The dorsal nasal meatus was most affected (green arrow). (F) Olfactory epithelial thickness differed significantly at 5 dpi: WT showed the most severe, OTS-206 moderate reduction, and OTS-228 preserved thickness (scale bars, 50 μm). Ordinary one-way ANOVA with Tukey’s multiple comparison was performed. Multiple virus variant comparisons (WT vs. OTS-206 vs. OTS-228) were performed at each dpi separately (2 or 5 or 21 dpi), and multiple time-point comparisons (2 vs. 5 vs. 21 dpi) were performed for each virus variant separately (WT or OTS-206 or OTS-228). ∗*p* < 0.05, ns = not significant. Mean with standard deviation is shown. Gray bar marks physiological limits. *n* = 5 hamsters per group; 2, 5, and 21 dpi; nasal levels 1, 2, and 3, with a total of 7 regions of interest were analyzed for the evaluation of the disorganization. The epithelial thickness was measured at the nasal septum at level 3.

**Figure 4:**
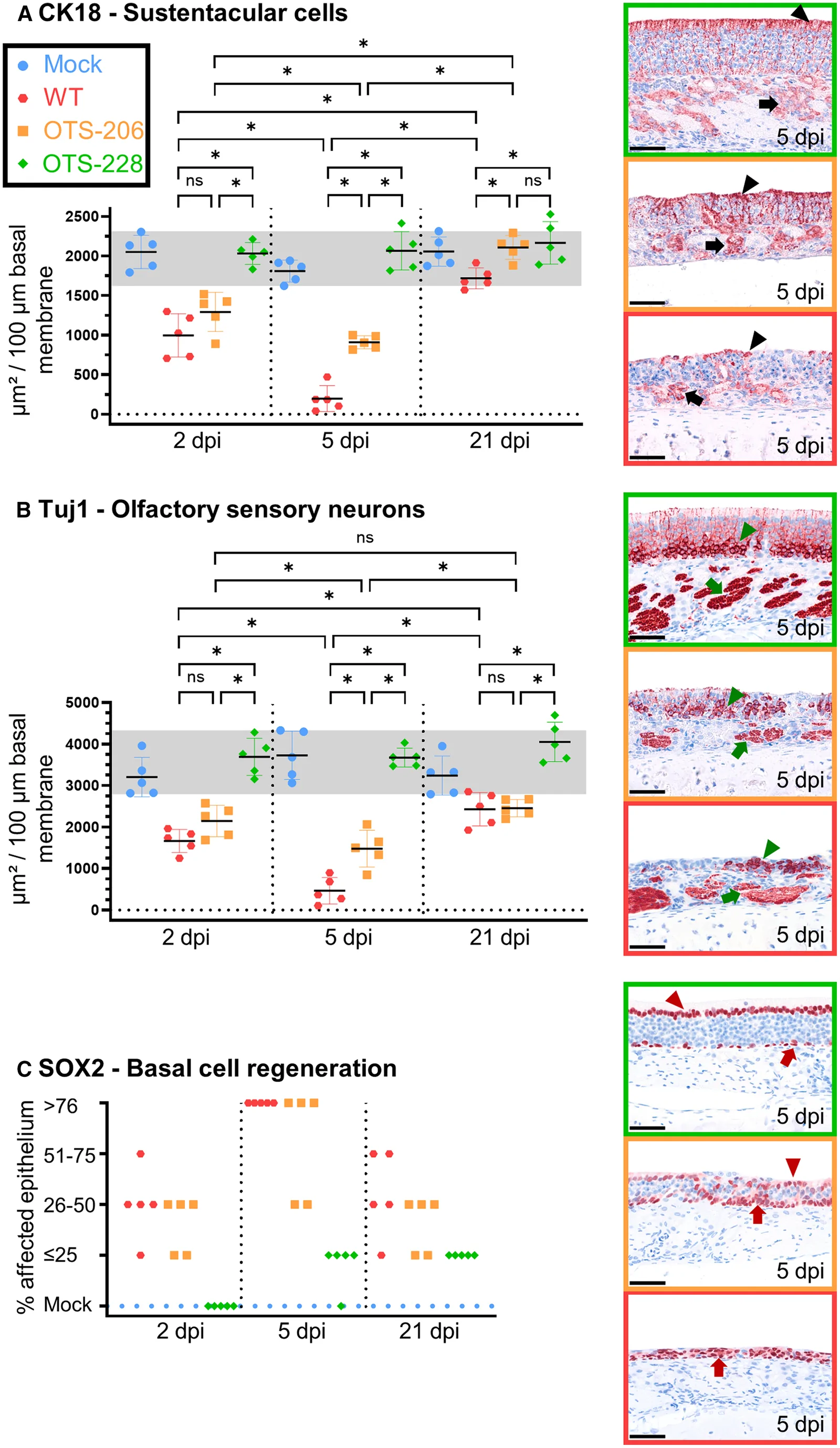
Loss of target and bystander cells and basal cell regeneration in the olfactory epithelium at 2, 5 and 21 dpi analyzed by digital image analysis (A) CK18 labeling of sustentacular cells (SCs) with almost complete loss after WT inoculation, up to 50% loss after OTS-206, and no changes after OTS-228 inoculation. At 21 dpi, SC area recovered to physiological levels in all groups. Representative CK18 labeled sections at 5 dpi show cytoplasmic labeling of SCs (black arrowhead) and Bowman’s gland epithelial cells (black arrow) (scale bars, 50 μm). (B) Tuj1 labeling of olfactory sensory neurons (OSNs) showed almost complete loss after WT and ∼50% loss after OTS-206 inoculation. OSN area remained below physiological levels at 21 dpi for WT and OTS-206, but was unchanged for OTS-228 at all time points. Representative Tuj1 labeled sections at 5 dpi show cytoplasmic labeling of OSNs (green arrowhead) and axon bundles (green arrow) (scale bars, 50 μm). Ordinary one-way ANOVA with Tukey’s multiple comparison was performed (A, B). Multiple virus variant comparisons (WT vs. OTS-206 vs. OTS-228) were performed at each dpi separately (2 or 5 or 21 dpi), and multiple time-point comparisons (2 vs. 5 vs. 21 dpi) were performed for each virus variant separately (WT or OTS-206 or OTS-228). ∗*p* < 0.05, ns = not significant. Mean with standard deviation is shown. Gray bar marks physiological limits. (C) SOX2 labeling of basal cells revealed pronounced hyperplasia in up to 100% of the olfactory epithelium after WT and OTS-206 inoculation, including multilayered basal cells in some regions. OTS-228 induced mild hyperplasia, affecting up to 25% of the olfactory epithelium. Representative SOX2 labeling at 5 dpi shows nuclei of basal cells (red arrow) and SCs (red arrowhead) (scale bars, 50 μm). CK18: cytokeratin peptide 18; Tuj1: neuron-specific class III beta-tubulin; SOX2: sex determining region Y box 2. *n* = 5 hamsters per group; 2, 5, and 21 dpi; nasal levels 1, 2, and 3, with a total of 7 regions of interest analyzed.

Detailed results for disorganization and the lesions description are provided in Figures 3A–3E for the olfactory epithelium and in Figures S2A–S2E for the respiratory epithelium, as well as in the Notes S2. Overall, the olfactory epithelium was more consistently affected than the respiratory epithelium. Briefly, in the olfactory epithelium, disorganization decreased from WT to OTS-206 to OTS-228, started at 2 dpi for WT and OTS-206, peaked for all variants at 5 dpi, and partially resolved with minimal residual changes persisting up to 21 dpi. In the respiratory epithelium, disorganization followed the same trend but was less pronounced and fully resolved by 21 dpi.

The thickness of the olfactory epithelium (Figure 3F) decreased most prominently in WT (≈ two-thirds at 5 dpi), less in OTS-206 (≈ one-third), both with full recovery by 21 dpi, and was unaffected in OTS-228. In the respiratory epithelium, thickness (Figure S2F) was reduced early in WT (from 2 dpi), but returned to physiological levels by 21 dpi, and remained largely unaffected in OTS-206 and OTS-228. For this parameter, statistically significant differences were identified at all time points for WT versus OTS-228 (olfactory and respiratory epithelium), between WT and OTS-206 (olfactory epithelium, 5 and 21 dpi; respiratory epithelium, 2 and 5 dpi), and even for OTS-206 versus OTS-228 (olfactory epithelium, 5 dpi).

Quantification of SC (Figure 4A) and OSN (Figure 4B) areas revealed that both were affected, although SCs are the primary target cells and statistically significant differences were detectable: WT caused early loss of both cell types (2 dpi), becoming nearly complete and significantly more pronounced than LAVs by 5 dpi. OTS-206 followed a similar but less severe pattern, with significantly greater loss than OTS-228 (2 and 5 dpi) but less than WT (5 dpi). By 21 dpi, SC areas recovered, whereas OSNs remained below physiological levels in WT and OTS-206. OTS-228 had no effect on either cell type.

Basal cell hyperplasia, indicating regeneration in the olfactory epithelium (Figure 4C), was evident for WT and OTS-206 from 2 dpi, peaked at 5 dpi, and declined by day 21. It was less pronounced for OTS-206 compared to WT, and only minimal and delayed after OTS-228 inoculation.

In summary, we observed a consistent gradient of tissue involvement across variants, with maximal damage in WT, intermediate effects in the moderately attenuated OTS-206, and minimal changes in the highly attenuated OTS-228. This was most pronounced in the olfactory epithelium, characterized by disorganization, thinning, and loss of SCs and OSNs, followed by basal cell-driven regeneration. The respiratory epithelium was less affected and recovered more rapidly. Notably, while SCs largely regenerated by 21 dpi, persistent OSN loss after WT and OTS-206 inoculation indicates incomplete functional recovery despite morphological restoration.

Interestingly, despite comparable extent of SARS-NP positive areas in the olfactory epithelium after WT and OTS-206 inoculation, tissue alteration differed markedly and was particularly limited for OTS-228. This gradual gradation was also already evident in the clinical (Figures S1B and S1C; Notes S1) and lung pathology data (Figure S3; Notes S1).

### Spatial and subcellular RNA analysis reveals distinct antiviral transcript localization patterns in infected and bystander cells

As indicated above, and not unexpected for viral infections, the severity of morphological alterations could not be fully explained by the extent of viral spread within the tissue. Infection may exert effects, e.g., on non-infected cells (bystander effects), as exemplified here by the loss of OSN area. We therefore aim to illustrate an approach to address such discrepancies using multiplex RNA ISH combined with antigen detection to analyze gene expression in defined cell populations and their microenvironment by confocal microscopy. Our results are based on the analysis of two animals per group, with six images evaluated per individual. Increasing biological or technical replicates is readily achievable due to standardized sampling and processing, depending on available resources. The method also enables software-based signal quantification, where warranted.

We aimed to better understand the differences in tissue alterations and examined the early antiviral response of infected epithelial cells and potential paracrine effects on bystander cells in the olfactory epithelium. RNA expression of interferon-beta 1 (IFN-β1), the interferon-stimulated genes retinoic acid inducible gene I (RIG-I) and myxovirus resistance protein 1 (Mx1) was analyzed in infected and bystander cells using multiplex ISH combined with SARS-NP antigen detection. Particular attention was given to RNA subcellular localization, as some viruses suppress host protein synthesis by retaining host mRNAs in the nucleus of infected cells, an effect not expected in bystander cells.^41^^,^^42^^,^^43^ To ensure analysis of intact epithelial cells with active RNA expression, olfactory epithelium was examined at 2 dpi for WT, OTS-206, and OTS-228 and on day 5 for OTS-228. Detailed results are presented and described in Figure 5. Briefly, IFN-β1, RIG-I, and Mx1 RNA were found in WT positive SCs with predominantly nuclear localization, but non-infected bystander cells showed cytoplasmic localization of RIG-I and Mx1 RNA (Figure 5A). Following OTS-206 inoculation, we found cytoplasmic localization of IFN-β1, RIG-I, and Mx1 RNA in infected SCs and RIG-I and Mx1 RNA in bystander cells (Figure 5B). OTS-228 showed no detectable RNA at 2 dpi and only cytoplasmic RIG-I and Mx1 RNA at 5 dpi (Figures 5C and 5D). Mock inoculated animals showed only minimal cytoplasmic RIG-I RNA in scattered epithelial cells but no IFN-β1 or Mx1 RNA (data not shown).

**Figure 5:**
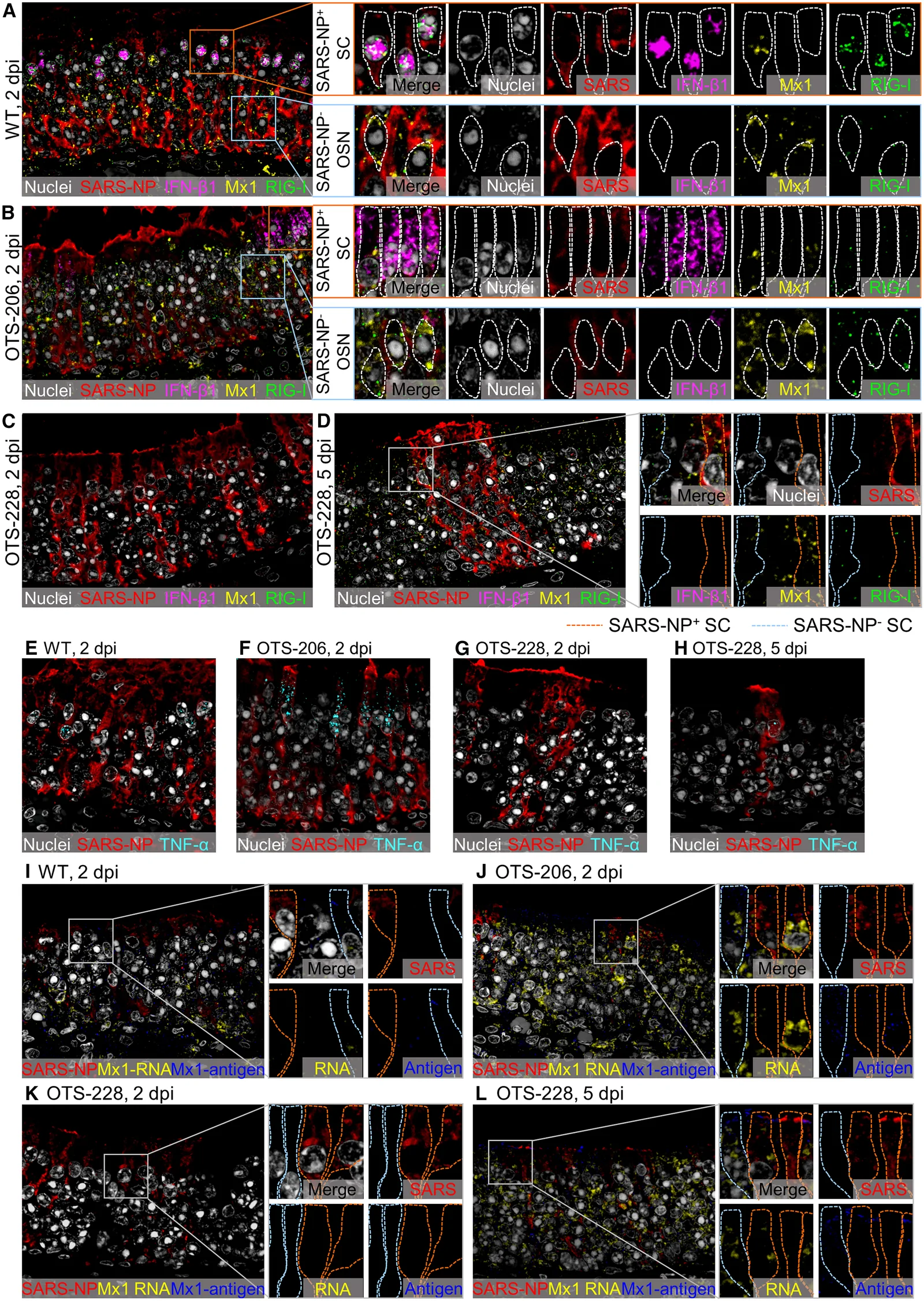
Host gene responses in the olfactory epithelium at 2 (WT, OTS-206, OTS-228) and 5 dpi (OTS-228 only). RNA *in situ* hybridization of interferon beta 1 (IFN β1), retinoic acid inducible gene I (RIG I), myxovirus resistance protein 1 (Mx1), and tumor necrosis factor alpha (TNF α) combined with SARS-NP and Mx1 antigen detection (A) WT: SARS-NP (red) positive sustentacular cells (SCs) showed mainly moderate to abundant nuclear IFN-β1 RNA (purple), few to moderate RIG-I (green), and few Mx1 (yellow) RNA signals. Non-infected olfactory sensory neurons (OSNs) exhibited few to moderate cytoplasmic RIG-I and Mx1 RNA. (B) OTS-206: SARS-NP positive SCs showed moderate to abundant cytoplasmic IFN-β1 RNA. Virus antigen positive SCs and negative OSNs exhibited moderate RIG-I and Mx1 RNA. (C and D) OTS-228: At 2 dpi (C), RNA expression of all targets was below the detection limit. At 5 dpi (D), IFN-β1 remained undetectable, whereas few to moderate cytoplasmic RIG-I and Mx1 RNA were detected in both SARS-NP positive and negative SCs. (E–H) TNF-α RNA (cyan) was present at moderate to abundant levels at 2 dpi after WT and OTS-206 inoculation and restricted to SARS-NP positive SCs, showing (E) nuclear aggregation in WT and (F) predominantly cytoplasmic distribution in OTS-206. (G and H) OTS-228: TNF-α RNA was undetectable at 2 dpi (G), but at 5 dpi (H), few cytoplasmic signals were present in individual SARS-NP positive SCs. (I–L) Mx1 RNA and antigen. (I) WT antigen positive SCs showed absent or few Mx1 RNA (yellow) signals and minimal protein (blue), whereas non-infected SCs exhibited low to moderate levels of both. (J) OTS-206 antigen positive and- negative SCs with moderate to abundant Mx1 RNA and antigen. (K and L) OTS-228 antigen positive cells with minimal Mx1 protein at 2 dpi (K), and moderate to abundant Mx1 RNA and protein at 5 dpi (L). Dotted contours indicate SCs and OSNs in magnified images. SARS-NP: SARS-CoV nucleocapsid protein. *n* = 2 hamsters per group; 2 (all groups) and 5 dpi (OTS-228 only); 6 images (63× magnification) per animal taken from regions of interest were evaluated for location (nuclear, cytoplasmic, both) and abundance of RNA signals (few, moderate, abundant).

Next, we analyzed TNF-α RNA expression, as TNF-α produced by epithelial cells has reported antiviral effects.^44^^,^^45^ As shown in Figures 5E–5H, TNF-α RNA was detected only in virus antigen positive SCs, again with mainly nuclear aggregation in WT (Figure 5E**)** and cytoplasmic localization in OTS-206-positive SCs (Figure 5F**)**. Following OTS-228 inoculation, only a few cytoplasmic TNF-α RNA signals were detected in individual SARS-NP positive SCs at 5 dpi (Figures 5G and 5H). TNF-α RNA was not detected in mock inoculated animals (data not shown).

In summary, spatial RNA analysis revealed distinct differences in subcellular localization of antiviral transcripts in both infected and non-infected bystander cells. WT infection was characterized by predominantly nuclear localization of RNA in infected cells, whereas both attenuated variants showed a more cytoplasmic distribution. This is consistent with altered host nuclear mRNA export, suggesting distinct host-virus interactions. Therefore, we next examined whether the predominantly nuclear RNA localization observed during WT infection could be associated with reduced host protein synthesis.

### Combined IHC and ISH to show virus-induced host shutoff through nuclear RNA retention

The combined IHC and ISH approach allows direct correlation of transcriptional and translational responses at single-cell level within intact tissue, providing functional insights into viral interference with host processes, such as protein synthesis, which is particularly relevant for viral pathogenesis. Based on the current findings and due to the availability of antibodies for hamster tissue, we applied combined Mx1 RNA and Mx1 protein detection and further included SARS-NP antigen labeling to enable analysis of infected cells only. Two animals per group and six images per animal were examined.

Detailed results are shown in Figures 5I–5L. WT showed limited Mx1 antigen in infected SCs, but up to moderate levels in bystander epithelial cells (Figure 5I). OTS-206 induced low to moderate levels of Mx1 antigen in infected SCs and moderate to abundant levels in adjacent epithelial cells (Figure 5J). OTS-228 showed minimal expression at 2 dpi, increasing by 5 dpi to levels comparable to OTS-206 at 2 dpi (Figures 5K and 5L).

In summary, WT infection of epithelial cells showed that predominantly nuclear Mx1 RNA localization was associated with a lack of or only minimal Mx1 protein detection, whereas non-infected bystander cells displayed up to moderate protein labeling. By contrast, both attenuated variants exhibited a predominantly cytoplasmic distribution of Mx1 RNA, accompanied by increased protein detection. Such differences in the early epithelial antiviral response may influence downstream effects, such as immune cell recruitment.

### Structured quantification reveals that the amount of immune cell infiltration does not necessarily correlate with tissue damage

Comparative quantification of infiltrating immune cells is per se relevant to the study of viral pathogenesis. To gain a deeper understanding of the association of immune cell response, virus spread, and tissue lesions, we detected immune cell infiltrates in the propria and epithelium of the olfactory and respiratory mucosa at 2, 5, and 21 dpi within the seven predefined ROIs, using serial consecutive sections. Iba1 (macrophages), CD3 (T-cells), and PAX5 (B-cells) IHCs were performed, and the area of macrophages (Iba = cytoplasmic signal) and the number of T-cells (CD3 = membranous signal) were quantified by digital image analysis, normalized to 100 μm of basal membrane. Due to the lack of a suitable cell marker for IHC, granulocyte infiltration was assessed semi-quantitatively following manual evaluation. In comparison to consecutive sections, the results could be interpreted in the context of viral antigen distribution and pathologic changes.

Following WT and OTS-206 inoculation, there is a reduction in epithelial thickness, which decreases the area of the epithelial surface that can be infiltrated by immune cells. Therefore, these data cannot be compared with those for OTS-228 and can be found in Figure S4. We thus focus on the lamina propria, which is largely independent of infection and lesions, making it suitable for statistical analysis and group comparison.

The results for the lamina propria are shown and described in detail in Figure 6. Overall, Iba1 positive macrophage areas were markedly increased 2 days after WT and OTS-206 inoculation, significantly exceeding those after OTS-228 inoculation (Figures 6A and 6B). It was also possible to discriminate macrophages showing a rounded, amoeboid morphology, typical of activation, following WT and OTS-206 inoculation, but mainly ramified morphology, indicative of a resting phenotype, following OTS-228 inoculation (Figure 6A**)**. By 5 dpi, OTS-228 reached levels comparable to WT and OTS-206 at 2 dpi (Figures 6A and 6B). Notably, numerous activated macrophages were associated with only a few OTS-228 positive epithelial cells and minimal to mild lesions.

**Figure 6:**
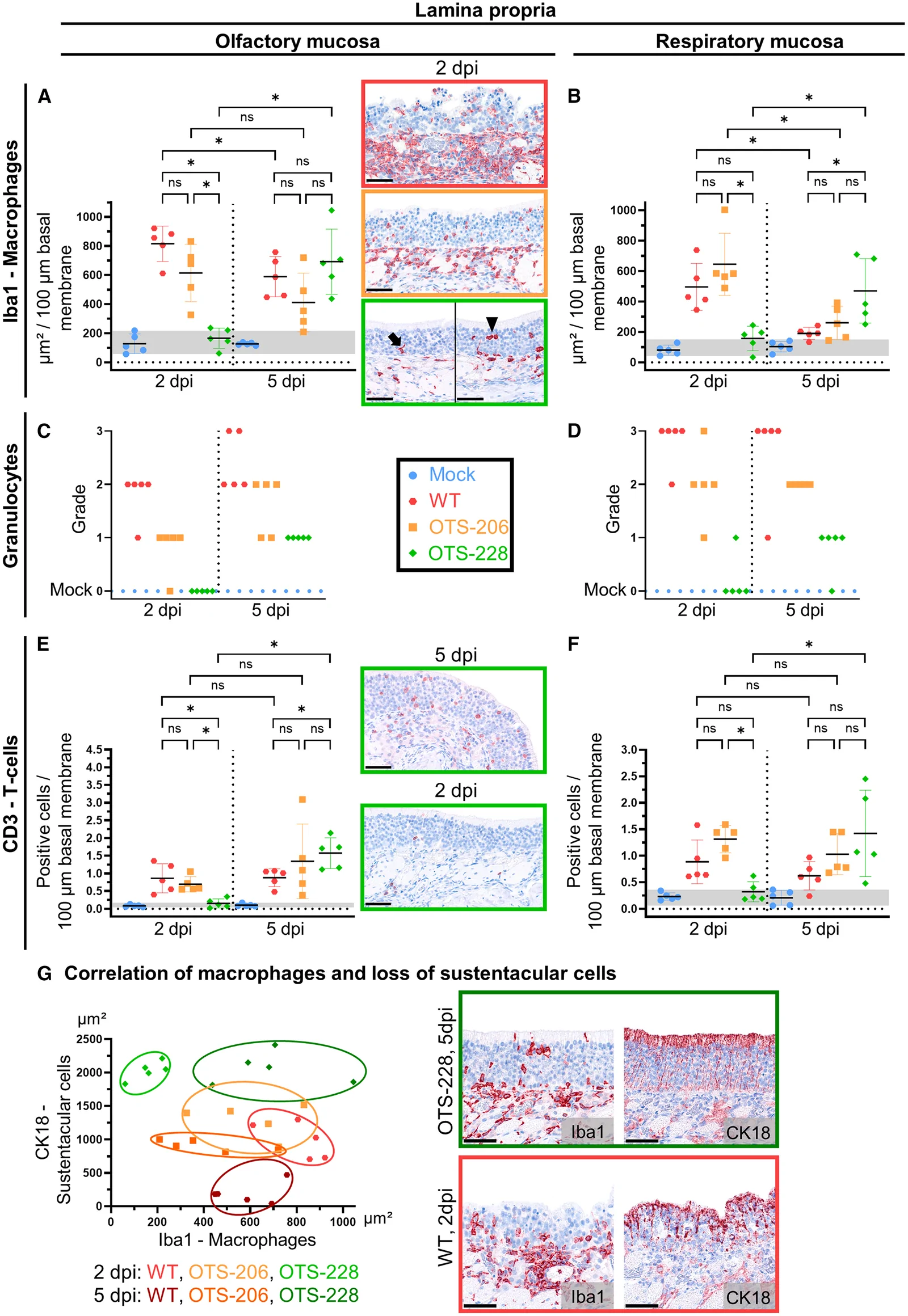
Immune cell infiltration in the lamina propria of the olfactory and respiratory mucosa at 2 and 5 dpi (A and B) Iba1 labeling with digital image analysis shows macrophage infiltration in the lamina propria of the olfactory (A) and respiratory mucosa (B). At 2 dpi, macrophage areas were significantly increased for WT (olfactory mucosa only) and OTS-206 compared to OTS-228. At 5 dpi, OTS-228 showed a delayed but significant increase, reaching levels comparable to WT and OTS-206. Representative Iba1 immunohistochemistry (IHC) at 2 dpi illustrates macrophage morphology: WT and OTS-206 showed predominantly rounded, amoeboid macrophages consistent with activation, whereas after OTS-228 inoculation most macrophages displayed a ramified morphology indicative of a resting phenotype (black arrow), with few activated macrophages (black arrowhead) (scale bars, 50 μm). (C and D) Granulocyte infiltration was semi-quantified, showing graded severity for WT and OTS-206, while OTS-228 induced only scattered granulocytes with minimal severity at 5 dpi. Higher grades were observed in the respiratory mucosa (D) compared to the olfactory mucosa (C). (E and F) CD3 labeling with digital image analysis showed T-cell infiltration in the olfactory (E) and respiratory mucosa (F). At 2 dpi, T-cell numbers were significantly higher for WT (olfactory mucosa only) and OTS-206 compared to OTS-228. At 5 dpi, OTS-228 showed a delayed but significant increase, reaching levels comparable to WT and OTS-206. Representative CD3 IHC for OTS-228 illustrates individual T-cells at 2 dpi and focal clusters of some T-cells at 5 dpi (scale bars, 50 μm). (G) Relationship between macrophage infiltration and sustentacular cell (SC) loss: WT and OTS-228 showed comparable macrophage areas, but only WT was associated with severe SC loss, whereas OTS-228 showed no SC damage (representative consecutive sections labeled for Iba1 and CK18, scale bars, 50 μm). For multiple group comparisons at 2 and 5 dpi, ordinary one-way ANOVA with Tukey’s multiple comparison (A and B) or Kruskal-Wallis test followed by Dunn’s correction (B, E, F) and for pairwise comparisons between time points, Welch’s (A, B) or Mann-Whitney (B, E, and F) test was performed. ∗*p* < 0.05, ns = not significant. Mean with standard deviation is shown. Gray bar marks physiological limits. Iba1: ionized calcium-binding adapter molecule 1; CD3: cluster of differentiation 3; CK18: cytokeratin peptide 18. *n* = 5 hamsters per group; 2 and 5 dpi; nasal levels 1, 2, and 3, with a total of 7 regions of interest analyzed.

Granulocyte infiltration largely correlated with viral antigen and tissue damage and reflected the gradual attenuation (Figures 6C and 6D). In general, infiltration was higher in the propria of the respiratory than in the olfactory mucosa. WT led most consistently to the highest severity scores, with disseminated (grade 2) to diffuse (grade 3) infiltration pattern, followed by OTS-206. After OTS-228 inoculation, some animals were affected but never exceeded minimal (grade 1) granulocytic infiltrates. By day 21, macrophage areas (Figures S5B and SBC) and granulocyte infiltration (Figures S5D and S5E) largely returned to physiological levels in all groups.

CD3 positive T-cell infiltration was overall low but largely mirrored macrophage findings in the propria of the olfactory and respiratory mucosa (Figures 6E and 6F). At 2 dpi, WT and OTS-206 induced significantly more T-cells than OTS-228. By day 5, OTS-228 led to a delayed but significant increase, reaching levels comparable to WT and OTS-206 at 2 dpi. By day 21, T-cell counts largely returned to physiological levels in all groups (Figures S5F and S1G).

PAX5 positive B-cells were absent or only sporadically detected in both olfactory and respiratory mucosa, regardless of virus variant or time point (data not shown).

Overall, in WT- and OTS-206 inoculated animals, large areas of macrophages were associated with many SARS-NP-positive epithelial cells and moderate to severe epithelial damage. In contrast, few OTS-228 positive cells with minimal lesions were associated with WT and OTS-206 comparable macrophage areas (Figure 6G**)**. In summary, immune cell infiltration does not directly correlate with tissue damage, as exemplified by substantial macrophage and T-cell infiltration after OTS-228 despite minimal epithelial lesions, suggesting functional differences of infiltrating macrophages or damage by other cell types, e.g., granulocytes.^22^

### Spatially defined analysis reveals macrophage cytokine response differs despite similar macrophage abundance

We demonstrated that multiplex RNA ISH combined with antigen detection enables *in vivo* gene expression analysis within functional tissue compartments while providing single-cell level resolution to discriminate infected target cells from non-infected bystander cells. The same method can be applied to study gene expression in particular immune cell subsets. We again exploited consecutive sections to selectively examine areas positive for viral antigen. Because macrophage abundance did not explain differences in tissue damage between virus variants, we now analyzed cytokine expression in infection-associated, activated macrophages. Potential differences may explain the varying degree of recorded granulocyte infiltration.^22^ To gain an initial understanding, we analyzed RNA expression of the pro-inflammatory cytokine interleukin-1β (IL-1β) and tumor necrosis factor-α (TNF-α), and the immune-regulatory cytokine transforming growth factor β (TGF-β), using multiplex ISH combined with Iba1 IHC for macrophage identification in the olfactory mucosa at 2 and 5 dpi.

Detailed results are provided for WT (Figures 7A and 7B), OTS-206 (Figures 7C and 7D), OTS-228 (Figures 7E and 7F), and mock inoculated hamsters for comparison (Figures 7G and 7H). In summary, for our model viruses, macrophage activation was dominated by TGF-β expression, whereas IL-1β and TNF-α showed a gradient from WT to OTS-206 to OTS-228. Despite comparable macrophage abundance, a more restrained pro-inflammatory response in OTS infected animals may underlie granulocyte infiltration and reduced tissue damage.

**Figure 7:**
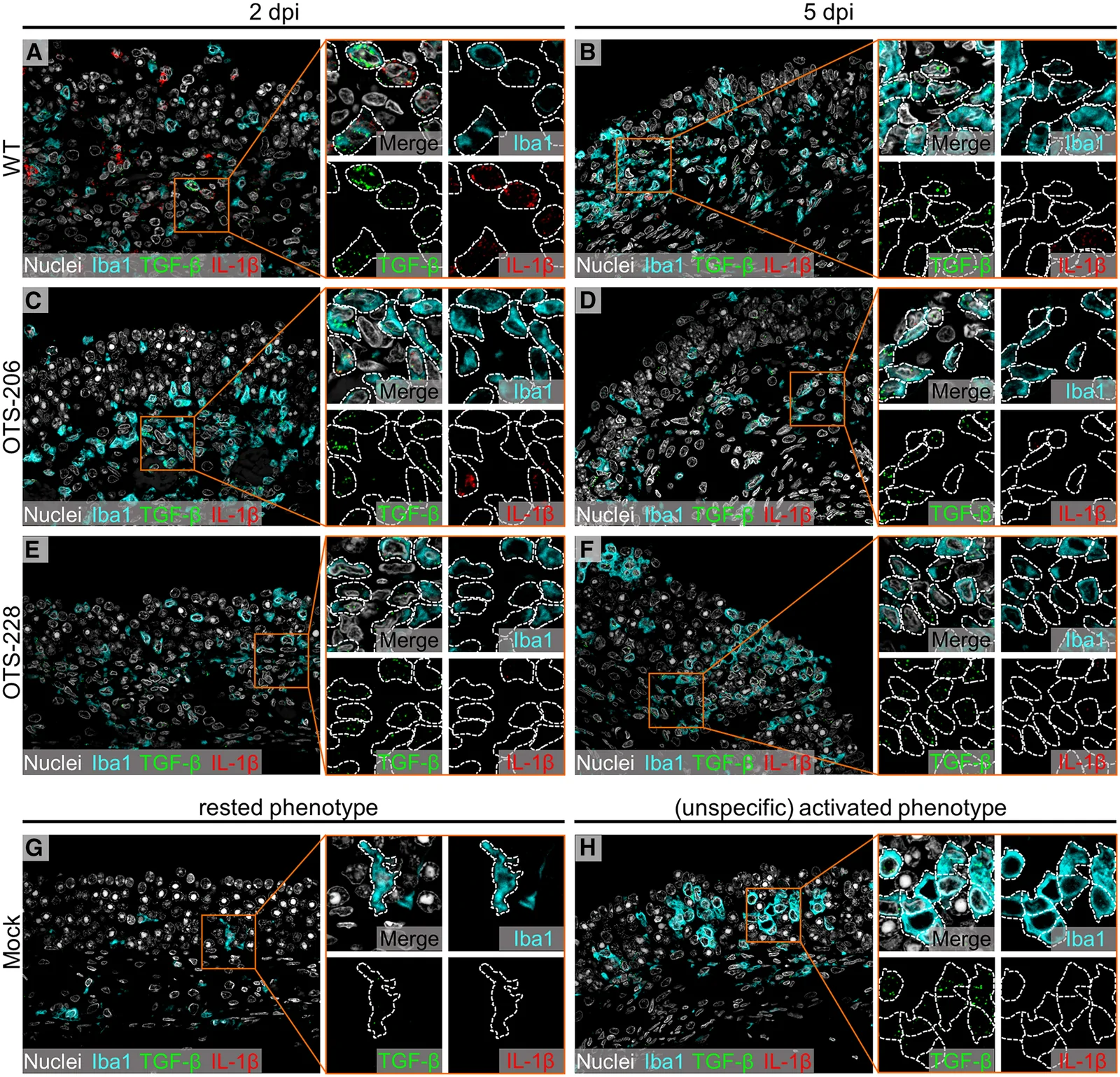
Lesion and infection-associated macrophage response at 2 and 5 dpi. RNA *in situ* hybridization of interleukin 1 beta (IL-1β), tumor necrosis factor alpha (TNF-α), and transforming growth factor β (TGF-β) in macrophages infiltrating the olfactory mucosa combined with Iba1 antigen detection (A and B) WT: At 2 dpi (A), most macrophages (Iba1, cyan) showed moderate to abundant TGF-β RNA (green), with many also exhibiting abundant IL-1β RNA (red). At 5 dpi (B), macrophages predominantly showed abundant TGF-β, while only a few exhibited moderate IL-1β RNA. TNF-α RNA was detected in few macrophages with up to moderate levels at 2 and 5 dpi (not shown). (C and D) OTS-206: At 2 and 5 dpi, most macrophages exhibited abundant TGF-β RNA. At 2 dpi (C), some macrophages showed moderate IL-1β RNA, whereas at 5 dpi (D), only a few had low IL-1β levels. TNF-α RNA was detected in very few macrophages with few signals (not shown). (E and F) OTS-228: At 2 (E) and 5 (F) dpi, the majority of macrophages showed moderate to abundant TGF-β RNA, while only a few exhibited low IL-1β RNA. Scattered macrophages with resting morphology showed few TGF-β signals. In severely damaged epithelial regions, some macrophages exhibited up to moderate IL-1β RNA at 5 dpi (not shown). TNF-α RNA was not detectable (not shown). (G and H) Mock: Most macrophages displayed a resting phenotype with few TGF-β RNA signals (G). Focal clusters of activated macrophages showed abundant TGF-β and absence of IL-1β and TNF-α RNA (H). White dotted contours indicate macrophages in magnified images. *n* = 2 hamsters per group; 2 and 5 dpi; 6 images (63× magnification) per hamster taken from regions of interest were evaluated for abundance of RNA signals (few/low, moderate, abundant/high) detected either in few, some, many, or most macrophages.

The combined multiplex ISH and IHC approach allowed this differential activation to be resolved at cellular resolution within its spatial context, distinguishing lesion- and infection-associated macrophages from macrophages in non-infected tissue areas *in situ*. Future studies should investigate other cytokines and granulocyte-attracting chemokines in order to clarify communication between different immune cell types, such as the recruitment of granulocytes by macrophages.

The key findings of the comparative analysis of our model viruses are summarized in the graphical abstract. Overall, OTS-228 shows high attenuation, even in the nasal mucosa with limited infection, and induces a delayed yet robust antiviral response, as well as immune-regulatory, tissue-conserving macrophage activation. In contrast to WT, both attenuated OTS variants enable nuclear RNA export, including interferons and ISGs. This results in a balanced immune response with reduced tissue damage.

Having established that our spatial framework captures viral antigen distribution, tissue damage, and immune responses across viruses with graded attenuation, we next asked whether it can also identify correlates of protection in a challenge setting.

### Proof-of-principle: Framework application on a vaccine efficacy study

We refined the framework using selected, image-based readouts that we deemed to be the most suitable for an objective comparison and being readily implementable and scalable. We then applied this framework to a vaccine efficacy study. Following intranasal OTS-228 or intramuscular bivalent mRNA vaccination and challenge infection with a heterologous SARS-CoV-2 variant (Figure S6A), we quantified viral antigen, epithelial thickness, SC and OSN area, macrophage and T-cell infiltration in the olfactory and respiratory mucosa (Figures S6B–S6K). In this setting, our framework showed that a single intranasal OTS-228 dose reduced nasal infection and preserved epithelial integrity, achieving protection and local immune responses comparable to two intramuscular mRNA doses.

## Discussion

The nasal mucosa is the primary replication site for many emerging respiratory viruses, but remains underexplored due to anatomical complexity and technical challenges.^1^^,^^2^^,^^7^^,^^8^^,^^9^^,^^10^ The aim of this study was to address this gap by establishing a practical and scalable framework for spatially resolved analysis of respiratory virus infections in the nasal cavity. Using wild-type SARS-CoV-2 and two live-attenuated vaccine (LAV) candidates, OTS-206 and OTS-228, as model viruses, we integrate standardized sampling, histopathology, immunohistochemistry (IHC), multiplex RNA *in situ* hybridization (ISH), and digital image analysis to characterize virus-host interactions and the local immune response across tissue, cellular, and subcellular levels.

To place our approach in biological context, we show that the high attenuation of OTS-228 is fully reflected in the nasal mucosa (see **graphical abstract**). Despite limited infection and minimal tissue damage, it induces a delayed but functional interferon response and a robust macrophage-dominated, tissue-preserving immune reaction. Further, OTS-228 showed a reduced cell-to-cell spread and altered spatial distribution without changes in target cell tropism. In contrast, the highly virulent WT and moderately attenuated OTS-206 caused pronounced viral infection, epithelial damage, earlier inflammation, and delayed regeneration. Notably, SARS-CoV-2 employs multiple immune evasion strategies that interact, complement, and reinforce each other,^46^^,^^47^ several of which were modified or removed in OTS-206 and OTS-228 (NSP1 disruption and ORF6-8 deletions).^37^ Given their complexity, individual effects of these viral proteins are difficult to assess *in vivo*, and the attenuation profile reflects the combined impact of all engineered changes. However, OTS-228 differs from OTS-206 exclusively by an additional deletion of the furin cleavage site (FCS). Consequently, the comparison between OTS-206 and OTS-228 specifically addresses the contribution of the FCS within an otherwise identical genetic background. Our data showed clear biological differences between OTS-206 and OTS-228, including reduced infection of the olfactory epithelium, lower viral spread, diminished epithelial damage or altered innate immune activation, and proved the high impact of the FCS on the performance of highly attenuated live vaccines *in vivo* at the primary site of infection. This confirms and broadens previous findings that deletion of the FCS substantially contributes to reduced infection, cell-to-cell spread, transmission, and virulence.^37^^,^^40^^,^^48^^,^^49^^,^^50^^,^^51^ In conclusion, our framework has demonstrated to be effective at identifying distinct attenuation profiles enabling mechanistic insights *in vivo*. The added value of this approach lies in its spatial resolution, linking infection to tissue architecture, cell types, and localized host responses, which are not captured by conventional qPCR or bulk transcriptomic readouts.^18^^,^^28^^,^^33^^,^^34^

While advanced spatial technologies such as multiplex imaging and spatial transcriptomics offer high molecular resolution, their application to complex tissues remains limited due to technical and financial constraints.^14^^,^^16^^,^^17^ In contrast, our framework is built on histopathological methods that are available in standard pathology laboratories, making it broadly accessible. Its modular design enables laboratories to select, modify, or add individual analytical modules based on their technical and financial capabilities and research objectives. Standardized tissue sampling and serial sectioning further allow numerous analyses to be performed on the same specimen panel, eliminating the need for additional animal experiments. This supports cost-effective initial analysis of larger sample sizes and the generation of robust datasets (routine HE stain and antigen quantification). Based on this, hypotheses can be refined and more complex, costly, high-resolution follow-up studies can be applied, as demonstrated for interferon and cytokine expression using multiplex ISH. The latter can be expanded to all samples and remain more cost-effective than advanced spatial technologies. In summary, this stepwise, data-driven approach enables progression from broader screening to detailed mechanistic analyses. High-resolution methods can be applied iteratively and, if warranted, extended to the complete cohort using serial sections, making the framework both adaptable and scalable.

Standardized sampling and processing are essential for reproducibility.^2^^,^^7^^,^^11^ Predefined regions of interest (ROIs) based on anatomical landmarks and biologically justified criteria improve comparability and enable integration of morphological, antigen-, and RNA-based data. Although guideline frameworks support consistent trimming and orientation,^2^^,^^12^ they are not routinely applied in infection studies.^21^^,^^22^^,^^23^^,^^24^^,^^25^ Consistent with known anatomical differences in the nasal mucosa,^8^^,^^9^^,^^10^ we therefore emphasize morphology-driven ROI definition, improving comparability across samples and experimental groups compared with ad hoc or antigen-focused analyses.^21^^,^^26^^,^^30^ Furthermore, the functional and physiological properties of the nasal epithelium,^8^^,^^52^^,^^53^ as well as virus-specific features are critical considerations for ROI selection. Therefore, ROIs should be selected based on standardized anatomical landmarks, published literature, and preliminary experimental data relevant to the specific biological question. Accordingly, different pathogens or study objectives may require different ROIs. However, the spatial characterization using the core modules (lesion characterization, viral distribution, immune cell infiltration, and host responses in infected, neighboring, or specific immune cell populations) is relevant to virtually all respiratory viral infections. This underscores the adaptability and applicability of the framework.

Building on this, consecutive formalin-fixed paraffin-embedded (FFPE) sections enable spatial correlation of multiple parameters and remain the standard in pathology due to their robustness, biosafety, and broad compatibility.^54^ While high-resolution spatial transcriptomics offers greater molecular depth,^17^^,^^55^ its applicability in infection models is often constrained by technical and biosafety requirements affecting RNA integrity.^56^

Within this framework, as in any morphology-based investigation of disease pathogenesis, an expert histopathological assessment is essential for identifying, interpreting, and contextualizing tissue alterations (from 2 to 21 dpi), selecting consistent ROIs and parameters, and integrating data within the same spatial context. Histopathological evaluation was time-consuming and required substantial training and experience. Complementary quantitative methods provided additional value. Although software-assisted epithelial thickness measurements were faster, they were less sensitive at certain time points, likely because tissue thickness reflects not only epithelial integrity, but also inflammatory changes such as edema and immune cell infiltration. By contrast, quantifying specific cell populations captured both early and late changes, making it the most sensitive and robust parameter in our setup. Notably, no single metric was sufficient, highlighting the need for combined approaches.

Extending this analysis, immunohistochemical detection of viral antigen provided spatial information complementing quantitative molecular assays. While PCR is highly sensitive, it cannot distinguish between infectious virus, residual inoculum, or degraded genome.^57^ In contrast, antigen detection localizes infection to specific compartments and cells,^21^^,^^58^^,^^59^ but does not prove active replication,^60^ underscoring the need for integrated readouts.^5^^,^^18^^,^^61^

Digital image analysis provides more objective and consistent results and enables a far more scalable quantification than conventional visual evaluation by pathologists, thereby reducing inter-observer variability and allowing more detailed discrimination between gradually attenuated viruses.^62^^,^^63^ However, variability in software, parameters, and normalization remains challenging.^64^^,^^65^ Comparative analyses across different digital platforms showed that standardized workflows produce highly comparable results regardless of the software used.^66^ Normalization to tissue area can be confounded in inflamed tissues,^26^^,^^32^^,^^67^ whereas our use of basal membrane length provided a more stable and accurate anatomical reference. Marker-specific localization (cytoplasmic, membranous, nuclear) and cell morphology further require tailored analysis strategies.^65^ Therefore, some cells can be quantified by absolute cell count, while for others, measuring their total area is more appropriate.

Adding a molecular layer, spatial RNA analysis by ISH enabled assignment of gene expression at the tissue and cellular level to defined anatomical compartments and cell types. Compared with bulk approaches, the strength of this approach lies in its ability to preserve the spatial tissue context, while remaining more accessible than high-dimensional spatial transcriptomics.^16^^,^^17^ This allows for the targeted analysis of cytokine expression in immune cells associated with infection and lesions within defined anatomical structures.

Importantly, combining these tissue-based approaches required selection of appropriate epithelial regions and time points. In our study, the analyses were restricted to early time points (2 dpi) for epithelial RNA analysis, as later stages were compromised by tissue loss. Integration of RNA detection with antigen co-localization further allowed discrimination between infected and bystander cells within the same anatomical context, thereby enabling the analysis of antiviral communication and interaction between neighboring cells.

Finally, at the subcellular level, RNA localization provided insights into viral immune evasion by illustrating that IFN and ISG transcripts are induced but are predominantly nuclear in WT infected epithelial cells, whereas both LAVs exhibited a more cytoplasmic distribution. These subcellular patterns are consistent with differences in nuclear mRNA export and may contribute to divergent translational outcomes. Such *in vivo* spatial analyses support *in vitro* findings about the mechanisms of nuclear mRNA retention, attributable to the functions of NSP1 and ORF6.^68^^,^^69^^,^^70^ This highlights how *in vivo* spatially resolved analysis of the intact nasal mucosa can help reconcile discrepancies between transcriptional and protein-level data.

In conclusion, we present a modulatory, scalable and adaptable framework for spatially resolved analysis of respiratory virus infections, enabling standardized and quantifiable assessment of infection, target cells, tissue damage, immune responses, regeneration, and gene expression at the primary site of viral infection. The approach provides a versatile strategy to address key challenges in studying the nasal mucosa by integrating spatial pathology with molecular analyses to investigate infection dynamics and host responses. The main advantage is that all datasets can be analyzed within the same spatial context, which allows for a context-dependent interpretation of molecular, cellular, and tissue-level readouts.

Applied to SARS-CoV-2 and vaccine variants with different attenuation levels, this approach reveals graded infection and immune responses, and provides insights into virus-host interactions that are not accessible by conventional virological or bulk transcriptional analyses.

### Limitations of the study

Several limitations of this study should be acknowledged. First, our framework has not been formally validated with respect to inter-observer or inter-laboratory reproducibility, which would require a systematic evaluation of all workflow components, pre-analytical and analytical. Nevertheless, we minimized pre-analytical and analytical variability through standardized tissue sampling and processing, predefined anatomically based ROIs, validated staining protocols with appropriate controls, identical image acquisition settings, and expert review of all histopathological assessments and tissue annotations.

Second, cell-segmentation-based quantification of ISH signals was not carried out due to anatomical and methodological constraints: i) membrane markers for segmentation could not be incorporated into the multiplex staining, because we were limited to using a single antigen marker to identify infected cells or macrophages; ii) the complex architecture of the pseudostratified olfactory epithelium and the variable morphology of macrophages precluded cell segmentation based on nuclear expansion; iii) colocalization of diffuse antigen signals with punctate RNA signals was also not suitable, as it would have excluded the bystander cells and introduced variability due to heterogeneous antigen expression of Iba1 across different states of macrophage activation. Although cell-segmented quantification is feasible in other tissues or with appropriate marker panels, our ISH analyses therefore relied on a reviewed, semiquantitative scoring system. We also acknowledge that the small number of animals (*n* = 2 per group) limits statistical power for quantitative comparisons. For the mechanistic and spatially focused questions addressed here, however, this sample size was sufficient to demonstrate the approach’s capabilities.

Third, while techniques are widely available, accurate interpretation of nasal histopathology requires expertise, a prerequisite common to all morphology-based investigations of disease pathogenesis rather than a limitation specific to this framework. Indeed, the absence of appropriate pathology expertise has been associated with erroneous interpretation of histological findings.^71^

Last, multiplex ISH enables spatially resolved gene expression analysis, but the number of detectable transcripts remains limited compared with high-throughput approaches.

## Resource availability

### Lead contact

Requests for further information and resources should be directed to and will be fulfilled by the lead contact, Angele Breithaupt (angele.breithaupt@fli.de).

### Materials availability

Tissues used in this study are available upon request from the lead contact.

### Data and code availability

All data reported in this paper are available within the article, its supplementary information, or will be shared by the lead contact upon request. This paper does not report original code. Any additional information required to reanalyze the data reported in this paper is available from the lead contact upon request.

## Acknowledgments

We thank Robin Brandt, Silvia Schuparis and Mareen Grawe for their excellent technical assistance, Julia Sehl-Ewert for participation in our review process and fruitful discussions, and the animal caretakers for their dedicated work. This work was supported and funded by RocketVax AG.

## Author contributions

Conceptualization, T.B., A.B., J.S., D.H., M.B., B.C., and J.K.; methodology, T.B., and A.B.; data curation, T.B., A.B., and J.S.; formal analysis, T.B., and J.S.; investigation, T.B., J.S., and N.J.H.; validation, T.B., and A.B.; visualization, T.B., and A.B.; supervision, A.B.; project administration, M.B.; resources, M.B.; writing - original draft preparation, T.B., and A.B., and writing - review and editing, T.B., A.B., J.S., D.H., M.B., B.C., J.K., N.J.H., L.U., and R.K.

## Declaration of interests

The University of Bern has filed a patent application for the use of OTS-206 and OTS-228 as a vaccine. In this application, J.S., N.J.H., L.U., D.H. and M.B. are named as inventors. The Friedrich-Loeffler-Institute is collaborating with the University of Bern and RocketVax AG for the development of OTS vaccines and receives funding for research. The authors declare no potential conflicts of interest with respect to the research, authorship, and/or publication of this article.

## STAR★Methods

### Key resources table

**Table:** 

REAGENT or RESOURCE	SOURCE	IDENTIFIER
**Antibodies**		

Rabbit polyclonal anti-Sars Nucleocapsid Protein	Rockland	Cat# 200–401-A50; RRID: AB_828403
Mouse monoclonal anti Sars-N (clone: 4F3C4)	Friedrich-Loeffler-Institut, Lab Reiche; Bussmann et al.^72^	https://doi.org/10.1016/j.virusres.2006.07.005
Rabbit polyclonal anti-Iba1	FUJIFILM Wako Pure Chemical Corporation	Cat# 019–19741; RRID: AB_839504
Rabbit polyclonal anti-Human CD3	Agilent	Cat# A0452; RRID: AB_2335677
Rabbit monoclonal anti-PAX5 (clone: EPR3730(2))	Abcam	Cat# ab109443; RRID: AB_10862070
Mouse monoclonal anti-Beta-3 Tubulin (clone: 2G10)	Thermo Fisher Scientific	Cat# MA1-118; RRID: AB_2536829
Mouse monoclonal anti-Cytokeratin Peptide 18 (clone: CY-90)	Merck KGaA	Cat# C8541; RRID: AB_476885
Rat monoclonal anti-SOX2 (clone: Btjce)	Thermo Fisher Scientific	Cat# 14–9811-82; RRID: AB_11219471
Mouse monoclonal anti-RusV-CP (clone: 2H11B1)	Friedrich-Loeffler-Institut, Lab Reiche; Matiasek et al.^73^	https://doi.org/10.1038/s41467-023-36204-w
Rabbit monoclonal anti-RusV-CP (clone: 2H11B1)	Friedrich-Loeffler-Institut, Lab Reiche; Matiasek et al.^73^	https://doi.org/10.1038/s41467-023-36204-w
Goat Anti-Rabbit IgG (H+L), Biotinylated	Vector Laboratories	Cat# BA-1000; RRID: AB_2313606
Goat anti-Mouse IgG (H+L), biotinylated	Vector Laboratories	Cat# BA-9200; RRID: AB_2336171
Goat anti-Rat IgG (H+L), biotinylated	Vector Laboratories	Cat# BA-9400; RRID: AB_2336202
Goat anti-rabbit Alexa Fluor 568	Thermo Fisher Scientific	Cat# A-11011; RRID: AB_143157
Goat anti-mouse Alexa Fluor 488	Thermo Fisher Scientific	Cat# A-11001; RRID: AB_2534069
Goat anti-rabbit IgG (H+L), horseradish peroxidase conjugated	Vector Laboratories	Cat# PI-1000–1; RRID: AB_2916034
Anti-multi-species IgG, horseradish-peroxidase-conjugated	Innovative Diagnostics	SBVMILK ID Screen® Schmallenberg Virus Milk Indirect ELISA

**Bacterial and virus strains**		

SARS-CoV-2 WT^D614G^ BetaCoV/Germany/BavPat1/2020	kindly provided by Prof. Dr. Roman Wölfel	GenBank: EPI_ISL_406862
SARS-CoV-2 XBB.1.5 hCoV-19/Netherlands/NH-EMC-5667/2023	kindly provided by Prof. Dr. Bart L. Haagmans	GenBank: EPI_ISL_16640568
SARS-CoV-2 OTS-206	Schön et al.^37^	https://doi.org/10.1038/s41564-024-01755-1
SARS-CoV-2 OTS-228	Schön et al.^37^	https://doi.org/10.1038/s41564-024-01755-1

**Chemicals, peptides, and recombinant proteins**		

recombinant SARS-CoV-2 receptor-binding domain (RBD) antigen	Wernike et al.^74^	https://doi.org/10.1111/tbed.13926
Osteosoft™	Merck KGaA	Cat# 1017281000
35% Bovine serum albumin	Merck KGaA	Cat# A7979
Triton X-100	FerakBerlin	N/A
VECTASTAIN® Elite ABC-HRP Kit, Peroxidase	Vector Laboratories	Cat# PK-6100
AEC Substrate Kit	Abcam	Cat# ab64252
Aquatex®	Merck KGaA	Cat# 1085620050
Immu-Mount™	Epredia	Cat# 9990402
ProLong™ Gold antifade mounting medium	Thermo Fisher Scientific	Cat# P36930
Tween®-20	Merck KGaA	Cat# P1379
Opal TSA-DIG Reagent	Akoya Biosciences	Cat# OP-001007
1x Antibody Diluent/Block	Akoya Biosciences	Cat# ARD1001EA
Opal 520 Reagent	Akoya Biosciences	Cat# OP-001001
Opal 570 Reagent	Akoya Biosciences	Cat# OP-001003
Opal 620 Reagent	Akoya Biosciences	Cat# OP-001004
Opal 690 Reagent	Akoya Biosciences	Cat# OP-001006
Opal 780 Reagent	Akoya Biosciences	Cat# OP-001008
Diamidin-2-phenylindol (DAPI	ACD Bio-Techne GmbH	Cat# 323108
HOECHST 33258	Merck KGaA	Cat# ATEH99E40BB0

**Critical commercial assays**		

RNAscope Multiplex Fluorescent Detection Reagents v2	ACD Bio-Techne GmbH	Cat# 323110
RNAscope 4-Plex Ancillary Kit	ACD Bio-Techne GmbH	Cat# 323120
NucleoMag Vet kit	Macherey-Nagel	Cat# REF 744200.4
qScript XLT One-Step RT-qPCR ToughMix	QuantaBio	Cat# 95132–500

**Experimental models: Cell lines**		

Vero E6 cells	Friedrich-Loeffler-Institut (Collection of Cell Lines in Veterinary Medicine)	Cat# CCLV-RIE 0929

**Experimental models: Organisms/strains**		

Hamsters (Mesocricetus auratus) SPF Rj:AURA	Janvier Labs	Rj:AURA

**Oligonucleotides**		

SARS2-IP4-14059F (5′-GGT AAC TGG TAT GAT TTC G-3′)	Metabion	N/A
SARS2-IP4-14146R (5′-CTG GTC AAG GTT AAT ATA GG-3′)	Metabion	N/A
SARS2-IP4-14084 (5′-FAM-TCA TAC AAA CCA CGC CAG G-BHQ-3′)	Metabion	N/A
ACT-1030-F (5′-AGC GCA AGT ACT CCG TGT G-3′)	Metabion	N/A
ACT-1135-R (5′-CGG ACT CAT CGT ACT CCT GCT T-3′)	Metabion	N/A
ACT-1081-HEX (5′-HEX-TCG CTG TCC ACC TTC CAG CAG ATG T-BHQ1-3′)	Metabion	N/A

**Software and algorithms**		

Leica Application Suite X (version 4.7.0.28176)	Leica Microsystems	N/A
NDPview.2 plus (version 2.8.24)	Hamamatsu Photonics	N/A
HALO v4.0	Indica Labs	N/A
GraphPad Prism 10.4.1	GraphPad Software Inc.	N/A
BioRender.com	Biorender	N/A
Adobe Photoshop 12.0	Adobe Inc.	N/A

**Other**		

Mau-Polr2a-C1	ACD Bio-Techne GmbH	Cat# 890871-C1
Mau-Ppib-C2	ACD Bio-Techne GmbH	Cat# 890851-C2
Mau-Ubc-C3	ACD Bio-Techne GmbH	Cat# 890861-C3
Mau-Hprt1-C4	ACD Bio-Techne GmbH	Cat# 1848661-C4
RNAscope 4-plex Negative Control Probe	ACD Bio-Techne GmbH	Cat# 321831
Mau-Ifnb1-C1	ACD Bio-Techne GmbH	Cat# 1163061-C1
Mau-Rigi-C3	ACD Bio-Techne GmbH	Cat# 1848631-C3
Mau-Mx1-C4	ACD Bio-Techne GmbH	Cat# 1848641-C4
Mau-Tnf-C1	ACD Bio-Techne GmbH	Cat# 1062341-C1
Mau-Il1b-C2	ACD Bio-Techne GmbH	Cat# 1062331-C2
Mau-Tgfb1-C4	ACD Bio-Techne GmbH	Cat# 1848621-C4
Hamamatsu S60 scanner	Hamamatsu Photonics	N/A
Leica Stellaris 8 confocal laser scanning microscope	Leica Microsystems	N/A

### Experimental model and study participant details

#### Viruses and live-attenuated vaccines (LAVs)

The attenuated recombinant OTS vaccine candidates OTS-206 and OTS-228, which are based on wild-type SARS-CoV-2 virus (BetaCoV/Wuhan/IVDC-HB-01/2019, GenBank: MT108784), have been described in detail previously.^37^ Briefly, the yeast transformation-associated recombination cloning was utilized to integrate changes into the SARS-CoV-2 genome, targeting specific serine and leucine codons in the ORF1ab of the WT virus. This produced synonymous codons that are now only a single nucleotide different from stop codons.^35^^,^^36^ Further, two amino acid changes (K164A and H165A) in the NSP1 gene and deletions of the accessory genes ORF6, 7ab and 8, led to the OTS-206 construct.^37^ OTS-228 additionally featured a deletion of the polybasic spike S1/S2 furin cleavage site.^37^ For direct comparison with LAV, WT^D614G^ SARS-CoV-2 virus (BetaCoV/Germany/BavPat1/2020, GenBank: EPI_ISL_406862) was used. SARS-CoV-2 XBB.1.5 (hCoV-19/Netherlands/NH-EMC-5667/2023, GenBank: EPI_ISL_16640568) was selected for the challenge experiments after vaccination.

#### Animals

Specific-pathogen-free Syrian hamsters (*Mesocricetus auratus*), aged 5 weeks, were obtained from Janvier Labs (Le Genest-Saint-Isle, France). Equal numbers of females and males were used and housed in groups of up to three per individually ventilated cage under controlled environmental conditions (22–24 °C, 45–65% humidity) with a 12-h light-dark cycle for the circadian rhythm. Animals had *ad libitum* access to standard rodent chow and water, and were provided with enrichment materials such as straw and paper for nest building. Following an acclimatization period, baseline measurements, including body weight and general health assessments, were recorded. No further procedures were performed on the animals prior to the start of the experiment.

All hamster experiments were evaluated by the responsible ethics committee of the State Office of Agriculture, Food Safety, and Fishery in Mecklenburg-Western Pomerania (LALLF M-V) and gained governmental approval under registration number LVL MV TSD/7221.3-1-041/20. The experiments were performed in biosafety level 3 (BSL3) containment laboratories at the Friedrich-Loeffler-Institut, Greifswald-Insel Riems, Germany. The standard operating procedures of BSL3 facilities were approved by relevant authorities in Germany. All personnel received relevant training before commencing work in BSL3 laboratories.

Both sexes were included to maintain a representative cohort, but sex-specific analyses were not conducted. The study focused on establishing a spatially resolved methodological framework, rather than to investigate sex-dependent effects. The outputs were not expected to differ in a manner relevant to the methodological objectives of this work.

### Method details

#### First animal study: Evaluation of LAV attenuation

The study and all virological methods were carried out as previously established and described.^37^^,^^38^ The experimental design is depicted in Figure S1A.

Intranasal inoculation was performed using a micropipette to administer 35 μL per nostril (70 μL total) of 10^4^ TCID_50_/animal of the indicated WT^D614G^ SARS-CoV-2 virus or LAVs OTS-206 and OTS-228. The final tissue culture infectious dose 50% (TCID_50_) per animal was determined on Vero E6 cells and was 10^4.21^ TCID_50_/animal for WT virus, 10^4.4^ TCID_50_/animal for OTS-206 and 10^3.42^ TCID_50_/animal for OTS-228 per animal. Mock vaccinated animals received non-infectious cell culture supernatant. To quantify viral shedding, nasal washes were collected by flushing 200 μL PBS into each nostril and collecting the reflux in safe-lock tubes at indicated time points. Both inoculation and nasal washing were conducted under short-term open-drop isoflurane anesthesia, resulting in unconsciousness for less than one minute. Physical well-being and body weight were monitored daily using a precision scale (d = 0.1 g). A body weight loss of ≥20% post inoculation was defined as humane endpoint. Five animals per group were dissected for tissue sampling at 2, 5 and 21 days after inoculation (dpi). Euthanasia was performed via isoflurane overdose, followed by bleeding and decapitation. After infection with WT virus, all five animals meet human endpoint criteria before the intended endpoint of 21 dpi. For animal welfare reasons, five naive contact animals, introduced 1 dpi to donors and euthanized on day 20 post contact, were used for compensation. Based on published data, no significant effects on the analyses conducted on day 21 are to be expected from the replacement by contact animals.^27^^,^^58^ At dissection, organ samples (trachea, cranial, medial, and caudal right lung lobes) were taken for virus genome quantification and serum samples were obtained for serological testing. Nasal washes, organ and serum samples were stored at -80 °C. Heads and left lung lobes were obtained for histopathological evaluation.

##### Virus genome quantification

Viral nucleic acids were isolated from 100 μL of nasal washings or 100 μL of tissue/organ homogenate supernatant using the NucleoMag Vet kit (Macherey-Nagel) according to the manufacturer’s instructions. SARS-CoV-2 RNA was detected by one-step RT-qPCR targeting the RNA-dependent RNA polymerase (RdRp) gene using the Institut Pasteur IP4 primer/probe set. The primer sequences were SARS2-IP4-14059F (5′-GGT AAC TGG TAT GAT TTC G-3′) and SARS2-IP4-14146R (5′-CTG GTC AAG GTT AAT ATA GG-3′), together with the FAM-labelled probe SARS2-IP4-14084 (5′-FAM-TCA TAC AAA CCA CGC CAG G-BHQ-3′), which amplifies nucleotides 14080–14186 of the SARS-CoV-2 genome.

To monitor sample integrity and nucleic acid extraction, β-actin was amplified in parallel as an internal control using the primers ACT-1030-F (5′-AGC GCA AGT ACT CCG TGT G-3′) and ACT-1135-R (5′-CGG ACT CAT CGT ACT CCT GCT T-3′), together with the HEX-labelled probe ACT-1081-HEX (5′-HEX-TCG CTG TCC ACC TTC CAG CAG ATG T-BHQ1-3′). As the forward primer ACT-1030-F detects both genomic DNA and RNA, amplification of β-actin served to confirm successful sample collection and nucleic acid extraction.

RT-qPCR reactions were carried out using qScript XLT One-Step RT-qPCR ToughMix (QuantaBio) in a total reaction volume of 12.5 μL containing 1 μL of the FAM primer/probe mix and 2.5 μL of extracted RNA. Reverse transcription was performed at 50 °C for 10 min, followed by enzyme activation at 95 °C for 1 min. Amplification consisted of 42 cycles of 95 °C for 10 s, 60 °C for 10 s, and 68 °C for 20 s, with fluorescence acquisition during the annealing step. Reactions were run on a CFX96 Real-Time PCR Detection System (Bio-Rad). Viral genome copy numbers per milliliter were determined by comparison with a serial dilution of a quantified RNA standard previously calibrated by digital droplet PCR. A cut-off of 10^3^ genome copies/mL was used for data interpretation.

##### Virus titration

Infectious virus titers were determined by endpoint dilution assay on Vero E6 cells. Briefly, cells were seeded into 96-well plates 24 h prior to infection to obtain an 80–90% confluent monolayer. Samples were serially diluted 10-fold, and 100 μL of each dilution was added to the cells. Each dilution was tested in 16 technical replicates. Following incubation for 72 h at 37 °C in a humidified atmosphere containing 5% CO_2_, infectious virus titers were calculated as 50% tissue culture infectious doses (TCID_50_) using the Spearman–Kärber method.

##### RBD-specific enzyme-linked immunosorbent assay (RBD-ELISA)

RBD-specific antibodies were quantified by indirect enzyme-linked immunosorbent assay (ELISA) following a protocol adapted from a previously published method with demonstrated high sensitivity and specificity.^74^ Briefly, high-binding 96-well plates (Greiner, Kremsmünster, Austria) were coated overnight at 4 °C with 100 ng of recombinant SARS-CoV-2 receptor-binding domain (RBD) antigen per well in 0.1 M carbonate buffer (pH 9.6). Plates were subsequently blocked with 5% skim milk in phosphate-buffered saline (PBS) for 1 h at 37 °C. Serum samples were diluted 1:100 in Tris-buffered saline containing Tween 20 (TBST) and incubated on the coated plates for 1 h at 37 °C. Bound antibodies were detected using a multi-species horseradish peroxidase-conjugated secondary antibody (SBVMILK; ID Screen Schmallenberg Virus Milk Indirect ELISA, IDvet) diluted 1:80. After addition of tetramethylbenzidine (TMB) substrate (IDEXX), absorbance was measured at 450 nm using a Tecan microplate reader (Tecan Group Ltd., Männedorf, Switzerland). Plates were washed three times with TBST between each incubation step. Two technical replicates each of predefined positive and negative control sera were included on every plate. The cut-off for positivity is ≥0.3.

#### Second animal study: Challenge experiment

To compare the protective effects of the intranasal OTS-228 LAV with an intramuscular mRNA vaccine at the primary site of replication, nasal tissue samples from previously published animal experiments were analyzed.^37^^,^^75^ Syrian hamsters were challenged with 10^4.5^ TCID_50_/animal SARS-CoV-2 XBB.1.5, following a single intranasal OTS-228 dose of 10^4.7^ TCID_50_/animal or two intramuscular infections of a bivalent mRNA vaccine (Comirnaty, Original/Omicron BA.4-5 [5 μg/hamster]) according to the planned or recommended vaccination strategy. Non-vaccinated, SARS-CoV-2 XBB.1.5 challenged animals served as controls. Heads were obtained for histopathological evaluation from four animals per group, 2 and 5 days post challenge. The vaccination regime, sampling, and dissections are shown in Figure S6A. Detailed information on the experimental setup, virological and lung pathological results were published previously.^37^^,^^75^

#### Tissue preparation for histopathology

The left lung lobe and head were immersion-fixed in 10% neutral-buffered formalin for 7 days. Following fixation, the heads were decalcified using Osteosoft (Merck KGaA, Germany) for 21 days at room temperature (RT) with gentle shaking. Three predefined transverse sections (levels) of the nose were prepared based on anatomical landmarks,^12^ and described epithelial distribution.^7^^,^^11^ The levels, arranged from rostral to caudal, are defined as follows: Level 1 is located directly posterior to the upper incisors, predominantly lined with respiratory epithelium; level 2 is located at the level of the incisive papilla, contains approximately equal proportions of respiratory and olfactory epithelium and level 3 is located between the first and second palatal ridges, primarily lined with olfactory epithelium (Figure 1A). Subsequently, nasal tissue sections were decalcified for further 72 hours, washed for 24 hours in 10% neutral-buffered formalin, embedded in paraffin and sections of 3–4 μm thickness of the rostral cut surface were mounted on Super-Frost-Plus-Slides (Thermo Fisher Scientific, Germany), dried for 90 minutes at 60°C and stored at RT until further processing. To enable the spatial correlation of all parameters assessed by different histopathological methods, serial consecutive sections were prepared from each nasal section (Figure 1B). For light microscopical examination, hematoxylin and eosin (HE) staining was performed.^76^

#### Immunohistochemistry (IHC)

For IHC on nasal tissue, consecutive slides were dewaxed in xylene, followed by rehydration in descending graded alcohols and the standardized avidin-biotin-peroxidase complex (ABC) method was applied. Details are given in Table S1. Endogenous peroxidase activity was quenched by incubating the tissue slides with 3% hydrogen peroxide (Merck KGaA, Germany) in distilled water for 10 minutes at RT. Antigen retrieval was performed using heat-induced epitope retrieval in a pressure cooker for 20 minutes. Slides were transferred to a humid chamber. To block nonspecific antibody binding, the slides were incubated for 30 minutes at RT with goat normal serum, diluted in tris buffered saline (TBS) at a 1:2 ratio. Primary antibodies were diluted in TBS with 1% bovine serum albumin (BSA, Merck KGaA) and 0,3% Triton X-100 (FerakBerlin, Germany), and incubated overnight at 4°C. The following primary antibodies were used: SARS-CoV nucleocapsid protein (SARS-NP), ionized calcium-binding adapter molecule 1 (Iba1) targeting macrophages, cluster of differentiation 3 (CD3) targeting T-cells, paired box protein Pax-5 (PAX5) targeting B-cells, cytokeratin peptide 18 (CK18) targeting sustentacular cells, neuron-specific class III beta-tubulin (Tuj1) targeting olfactory sensory neurons, and sex determining region Y box 2 (SOX2) targeting basal cells of the olfactory epithelium and sustentacular cells. Between all following steps, slides were carefully washed with TBS. Detection was achieved using a biotinylated secondary antibody diluted in TBS, followed by incubating the slides with avidin-biotin-peroxidase complex solution (ABC, Vectastain Elite ABC Kit; Vector Laboratories, USA) each for 30 minutes at RT and exposure to a 3-amino-9-ethylcarbazole substrate (AEC substrate Kit, Abcam, UK). For PAX5 an ImmPRESS Polymer Kit (Vector Laboratories, USA) was used, followed by AEC substrate. The AEC reaction was stopped in distilled water under visual control. Slides were counterstained with Mayer’s hematoxylin and cover slipped with Aquatex (Merck KGaA). As a negative control, consecutive sections were labeled with an irrelevant antibody targeting the capsid protein of *rustrela virus* (clone 2H11B1, in house, generated by Sven Reiche^73^). Depending on the primary antibody applied, positive control slides from SARS-CoV-2-infected lung, lymphoid, or brain tissue from Syrian hamsters were included in each run.

#### Immunofluorescence

For immunofluorescence, nasal tissue slides were dewaxed, rehydrated and antigen retrieval was performed as described for IHC. Details are given in Table S2. Slides were transferred to a humid chamber and permeabilization was carried out by incubating in permeabilization buffer, containing phosphate buffered saline (PBS) supplemented with 0.2% gelatin and 0.25% alkylphenyl polyethylene glycol, for 10 minutes at RT. A blocking buffer, containing permeabilization buffer with 5% BSA, was applied for 1 hour at RT. Primary antibodies were pooled and incubated over night at 4°C in dilution buffer (blocking buffer diluted in PBS, containing 1% BSA). Subsequently, slides were washed in PBS. To assess target cell tropism within the olfactory epithelium, a primary antibody against SARS-NP was combined with either antibodies against CK18 or Tuj1. Slides were incubated with permeabilization buffer for 10 minutes at RT, before pooled fluorophore conjugated secondary antibodies diluted in dilution buffer were applied for 1 hour at RT. Between the following steps, slides were carefully washed with PBS. Nuclear counterstaining was performed with Hoechst 33258 (1:20.000; Merck KGaA) for 5 minutes at RT. A final wash step was performed using washing buffer (10 mM CuSO_4_ and 50 mM NH_4_Cl in distilled water) for 10 minutes at RT. Slides were mounted with Epredia Immu-Mount. Imaging was conducted using a confocal laser scanning microscope (Leica Stellaris 8, Leica Microsystems, Germany), and image processing was performed with LAS X Office software (Leica Application Suite X, version 4.7.0.28176, Leica Microsystems). At least 6 randomly selected images taken from ROIs with infected epithelial cells of randomly selected animals per group were evaluated at 63x magnification for colocalization of SARS-NP with CK18 or Tuj1.

#### Multiplex RNA *in situ* hybridization (ISH) with combined antigen detection

Multiplex RNA *in situ* hybridization was performed using the RNAscope Multiplex Fluorescent Detection Reagents v2 (#323110) and the RNAscope 4-Plex Ancillary Kit (#323120) from Advanced Cell Diagnostics (ACD Bio-Techne GmbH, Germany) according to the user manual (UM 323100). Details are given in Table S3. RNA probes were used, targeting hamster interferon-beta 1 (IFN-β1), retinoic acid inducible gene I (RIG-I), myxovirus resistance protein 1 (Mx1), tumor necrosis factor-alpha (TNF-α), interleukin-1-beta (IL-1β) and transforming growth factor β (TGF-β). The protocol “RNAscope Multiplex Fluorescent v2 Assay” was used and adapted as follows: Tissue preparation and pretreatment were carried out using the protease-free method. Sections were deparaffinized in xylene and rehydrated in 99% Ethanol and a hydrophobic barrier was drawn around the tissue samples, followed by incubation with hydrogen peroxide for 10 minutes at RT. Target retrieval was performed in a pressure cooker using RNAscope 1x Target Retrieval Reagent at 99 °C for 35 minutes. After equilibration in distilled water and incubation in 1x RNAscope Wash Buffer for five minutes at RT, sections were treated with RNAscope Manual PretreatPro Reagent in a preheated HybEZ oven at 40 °C for 35 minutes. Following washing in distilled water and incubation in 1x RNAscope Wash Buffer (five minutes each), RNA probe hybridization was performed for 2 h at 40 °C in the HybEZ oven. For hybridization, probes were pooled: C1 probes were ready-to-use (1x), while warmed C2, C3, and C4 probes (50x) were diluted 1:50 in C1. Following RNA probe hybridization, sections were stored overnight at RT in 5x saline sodium citrate. Amplification steps were carried out sequentially with RNAscope Multiplex FL v2 Amp 1 for 30 minutes, Amp 2 for 30 minutes, and Amp 3 for 15 minutes at 40 °C in the HybEZ oven. HRP-C1 signal development involved incubation with RNAscope Multiplex FL v2 HRP-C1 for 15 minutes, opal fluorophore (Akoya Biosciences, USA) for C1 probe labeling, diluted 1:500 in RNAscope Multiplex TSA Buffer for 30 minutes, and RNAscope Multiplex v2 HRP Blocker for 25 min, each at 40 °C in the HybEZ oven. Washes in 1x RNAscope Wash Buffer were performed between all steps. Signal development for HRP-C2 to HRP-C4 was performed analogously using the corresponding reagents and opal fluorophores. For subsequent antigen detection of SARS-NP, Iba1, Mx1 or SARS-N (in house, generated by Sven Reiche^72^) the protocol “RNAscope Multiplex Fluorescent v2 Assay combined with Immunofluorescence” was used and adapted as follows: Sections were washed in TBS with 10% Tween-20 (TBST, Merck KGaA) and blocked with 10% normal goat serum in TBS-0.1% BSA for 30 minutes at RT. Subsequently, primary antibodies, diluted in TBS-0.1% BSA, were applied for 1 hour and 15 minutes at RT. Between the following steps, slides were washed with TBST. Slides were incubated with HRP-conjugated or fluorophore conjugated secondary antibodies, diluted 1:200 in TBS-0,1% BSA for 30 minutes at RT. Signal development of the HRP-conjugated antibody was achieved by stepwise incubation with Opal TSA-DIG Reagent (Akoya Biosciences) diluted 1:250 in RNAscope Multiplex TSA Buffer for 10 minutes at RT, RNAscope Multiplex FL v2 HRP Blocker for 25 min at 40 °C in the HybEZ oven, and Opal 780 Reagent (Akoya Biosciences) diluted 1:62,5 in 1x Antibody Diluent/Block (Akoya Biosciences) for 10 min at RT. Alternatively, the secondary antibody was labeled with Opal 520 Reagent (Akoya Biosciences) for 10 min at RT without prior incubation with TSA-DIG and HRP Blocker. Finally, sections were counterstained with Diamidin-2-phenylindol (DAPI, ACD Bio-Techne GmbH) for 30 seconds at RT and mounted directly using ProLong Gold antifade mounting medium (Thermo Fisher Scientific, USA). As negative and positive controls, archived nasal sections from SARS-CoV-2 positive hamsters were used. For antigen detection control, an irrelevant antibody (capsid protein of *rustrela virus*, clone 2H11B1, anti-rabbit 1:835, or anti-mouse 1:100, in house, generated by Sven Reiche^73^) was included. For RNAscope, a 4-plex negative control probe was used and positive control probes were applied targeting hamster RNA polymerase II subunit A, peptidylprolyl isomerase B, hypoxanthine phosphoribosyltransferase 1, and ubiquitin C. Imaging was conducted using a confocal laser scanning microscope (Leica Stellaris 8, Leica Microsystems), and image processing was performed with LAS X Office software (Leica Application Suite X, version 4.7.0.28176, Leica Microsystems). The acquisition settings on the microscope were adjusted for each experiment based on the positive and negative controls and applied consistently for each image. For each question, at least 6 randomly selected images taken from ROIs of randomly selected animals per group were evaluated at 63x magnification.

#### Evaluation strategy on whole slide digital images

For light microscopical examination slides were scanned with a Hamamatsu S60 scanner (Hamamatsu Photonics). Whole slide digital images were analyzed using NDPview.2 plus (version 2.8.24, Hamamatsu Photonics) and HALO software (HALO v4.0, Indica Labs). The initial histopathological diagnoses, annotations and image analysis were performed by a trained pathologist (TB) in a masked fashion using the post-examination masking method,^29^ and peer reviewed by an ECVP board-certified veterinary pathologist (AB, DiplECVP since 2015). In cases of discordant interpretations, the findings and annotations were discussed during a weekly consensus review meeting involving TB, AB, and an additional ACVP board-certified veterinary pathologist (DiplACVP). Final diagnoses and annotations were established by consensus. Finally, the overall framework data were reviewed by aDiplACVP (RK) who specializes in digital pathology.

##### Quantification of cell markers and viral antigen in the nasal mucosa

To ensure uniformity in the analysis of the nasal conchae in all 3 levels, regions of interest (ROIs) were predefined for histological evaluation and digital image analysis. Standardized anatomical landmarks,^7^^,^^11^^,^^12^ published SARS-CoV-2 data,^8^^,^^10^ and preliminary investigations from unpublished, archived experimental SARS-CoV-2 samples served as a basis for ROI selection, identifying anatomical regions that are consistently recognizable across animals and time points and encompass the major SARS-CoV-2-susceptible tissues. The following 7 ROIs were defined: Levels 1 and 2 = nasal septum and dorsal nasal meatus; level 3 = nasal septum, endoturbinate 1, and the dorso-medial surface of ectoturbinate 1 (Figure 1A).

Due to expected differences in the staining intensity, prior to digital image analysis, all tissue sections were categorized based on chromogen signal intensity and background labeling: Category 1 = strong chromogen signal with absent or minimal background, category 2 = weak chromogen signal with no background and category 3 = strong chromogen signal with pronounced background. This enabled i) consistency in analyzing sections within a category using identical parameters, ii) minimal adjustment of parameters to ensure plausible results when comparing samples with different staining intensity. Within the defined ROIs, the basal membrane, epithelium, and lamina propria were annotated and the length of the basal membrane was measured. False-positive, non-specific or background signals were excluded from analysis.

For SARS-NP, Iba1, Tuj1, and CK18 with diffuse, cytoplasmic labeling, the “Area Quantification v2.4.9 module” was used to determine the total area of positive pixels in μm^2^. A detailed list of parameters for each antibody and category, along with a detailed description of how to set these parameters, is provided in Table S4.

For CD3, with membranous labeling, the “Multiplex IHC v3.4.9 module” was used to count the total number of positive cells. A detailed list of parameters for each category, along with a detailed description of how to set these parameters, is provided in Tables S5 and S6.

All parameters were established within the same category of a specific antibody on five sections and validated on further five sections before the image analysis was performed. The optimal parameters remained unaltered following the commencement of the analysis. Following analysis, every fourth section and outliers were checked for accuracy.

The basal membrane was set as reference parameter, representing a consistent structural feature across all samples. The basal membrane length, the positive area (in μm^2^) of SARS-NP, Iba1, Tuj1, and CK18, as well as the absolute numbers of CD3 positive cells, were recorded separately for the epithelium and propria of the olfactory and respiratory mucosa, respectively. Finally, all values were calculated per 100 μm of basal membrane length. Measurements in mock inoculated animals served for the definition of the physiological range.

##### Analysis of morphological changes in the nasal mucosa

A detailed morphological examination of the respiratory epithelium and olfactory epithelium at 2, 5 and 21 dpi were conducted. Mock infected animals served as the reference baseline. First, histopathological findings were described. Further, based on HE stained slides, disorganization of the epithelial layer, and granulocyte infiltration was graded within the previous described seven ROIs and epithelial thickness was measured. Epithelial disorganization (disruption of the epithelial cell stratification, irregular luminal surface of the epithelium, loss of the physiological cell morphology and ciliary layer) was graded in a metric scale as follows: No disorganization (within physiological limits); disorganization of the epithelial layer affecting ≤25% (minimal), 26–50% (mild), 51–75% (moderate) or >76% (severe). Granulocyte infiltration was assessed separately for the epithelium and propria in an ordinal scale: Grade 0 = within physiological limits, grade 1 = minimal infiltration with only scattered granulocytes, grade 2 = moderate infiltration with disseminated granulocytes, grade 3 = diffuse infiltration by numerous granulocytes. Due to significant variability in epithelial thickness across the nasal mucosa,^9^ a particular ROI was defined and measurements were taken at the nasal septum in level 1 for the respiratory epithelium and level 3 for the olfactory epithelium. Bilaterally, 1.5 mm of the basal membrane and the overlying epithelium were annotated excluding cilia. Using the HALO tool “layer thickness”, epithelial thickness was measured every 50 μm, the mean thickness was calculated and used for comparison. Additionally, using basal cell labeling by SOX2 IHC, basal cell hyperplasia in the olfactory epithelium was scored in the same way as epithelial disorganization: Within physiological limits or hyperplasia of basal cells affecting ≤25%, 26–50%, 51–75% or >76% of the evaluated epithelium.

##### Analysis of multiplex RNA ISH with antigen colocalization

To analyze the early antiviral immune response of infected and adjacent uninfected cells in the olfactory epithelium, we performed RNA ISH on IFN-β1, RIG-I, Mx1, and TNF-α combined with SARS-NP antigen detection by IHC. Samples from 2 dpi for WT, OTS-206, OTS-228 and on 5 dpi for OTS-228 were included (well preserved tissues only). For comparison, mock inoculated animals were included. At least 6 images taken from ROIs of randomly selected animals per group were evaluated for location (nuclear, cytoplasmic, both) and abundance of RNA signals (few, moderate, abundant).

To better understand the macrophage response, we selected mucosal regions with particular virus antigen associated, activated macrophages, analyzed IL-1β, TNF-α, and TGF-β RNA combined with Iba1 IHC for macrophage identification in the olfactory mucosa at 2 and 5 dpi for WT, OTS-206 and -228. For comparison, mock inoculated animals were included. Analysis was performed on at least 6 images taken from ROIs of randomly selected animals per group, evaluated for abundance of RNA signals (few/low, moderate, abundant/high) detected either in few, some, many, or most cells.

##### Analysis of morphological changes and viral antigen in the lungs

In accordance to previous studies,^37^^,^^38^ the lungs from hamsters inoculated with OTS-206, OTS-228, WT SARS-CoV-2 and non-infectious cell culture supernatant at 5 dpi were evaluated using a 500 × 500 μm grid, and the extent of pneumonia-associated atelectasis was recorded as a percentage of the affected lung fields. Furthermore, the lungs were examined for the presence of SARS-CoV-2 characteristic lesions described for hamsters.^77^^,^^78^ These included intra-alveolar, interstitial, peribronchial and perivascular inflammatory infiltrates, alveolar edema, necrosis of the bronchial epithelium, diffuse alveolar damage, vasculitis, activation of endothelium with immune cell rolling, as well as bronchial epithelial or pneumocyte type 2 hyperplasia. SARS-NP (1:3000, Rockland, 200–401-A50) IHC was performed as described above. The distribution of virus antigen was graded on an ordinal scale. The scores were as follows: 0 = no antigen; 1 = focal, affected cells/tissue < 5% or up to 3 foci per tissue; 2 = multifocal, 6%–40% affected; 3 = coalescing, 41%–80% affected; 4 = diffuse, >80% affected. The target cell was identified based on its morphological characteristics.

### Quantification and statistical analysis

#### Statistics and visualization

Data visualization and statistical analysis were performed using GraphPad Prism 10.4.1 (GraphPad Software Inc., USA). Data are presented as mean with standard deviation, with each dot representing an individual hamster. Normal distribution was determined graphically using a Q-Q plot. For normally distributed datasets, the ordinary one-way or two-way ANOVA with Tukey’s multiple comparison for multiple group comparisons or Welch’s *t* test for pairwise comparisons were conducted. For datasets that were not normally distributed, the Kruskal-Wallis test followed by Dunn’s correction for multiple group comparisons or Mann-Whitney test for pairwise comparisons were performed. *p* < 0.05 was set as statistically significant. To ensure comparability, statistical tests were performed on epithelial datasets only for groups and time points that showed sufficiently preserved epithelium. Experimental setup and illustrations were created with BioRender.com. All histopathological images were uniformly adjusted for image levels using Adobe Photoshop 12.0.

## Supplemental information

Document S1. Figures S1–S6, Tables S1–S6, and Notes S1 and S2
