# Supplementary material for: A multimodal in situ framework for spatial characterization applied on SARS-CoV-2 live-attenuated vaccines in the nasal mucosa
Source: iScience. 2026 Sep 22;29(10):117730. doi: 10.1016/j.isci.2026.117730 (PMC13675480; doi:10.1016/j.isci.2026.117730)
Supplement: Supplementary file 1 [file mmc1.pdf]

iScience, Volume *29*

Supplemental information

A multimodal *in situ* framework for spatial characterization applied on SARS-CoV-2 live-attenuated vaccines in the nasal mucosa

Tobias Britzke, Jacob Schön, Nico Joël Halwe, Lorenz Ulrich, Jana Kochmann, Björn Corleis, Robert Klopfleisch, Martin Beer, Donata Hoffmann, and Angele Breithaupt

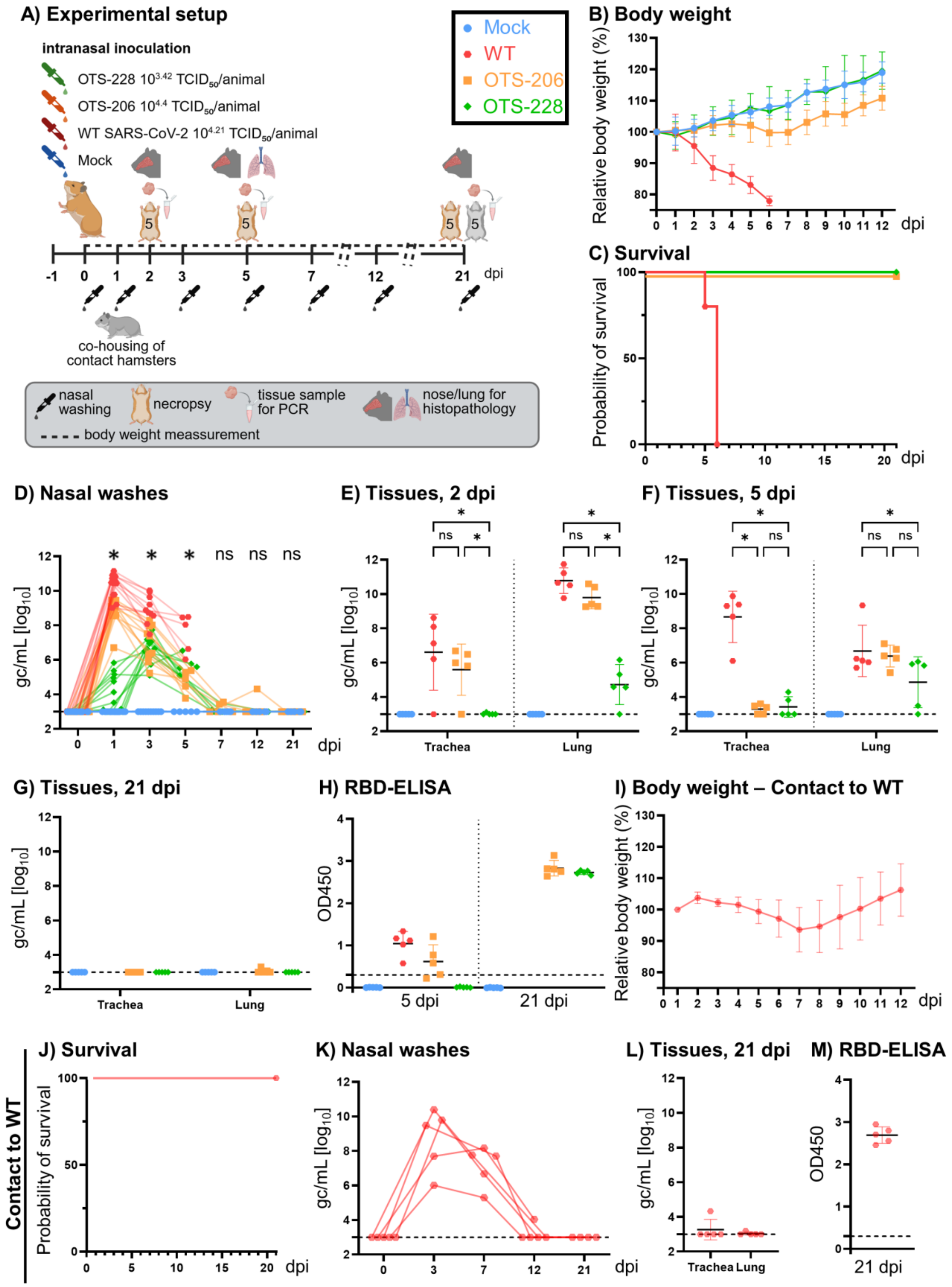

Figure S1: Experimental setup and virological data.

(A) Syrian hamsters (Mesocricetus auratus, female and male equally mixed, 5 weeks old) were intranasally inoculated with wild-type (WT) SARS-CoV-2, OTS-206 or OTS-228, Naïve contact animals were co-housed 1 dpi. Mock controls received non-infectious cell culture supernatant. Necropsies were performed at 2, 5, and 21 dpi; trachea and lung samples were collected for RT-qPCR, and head and left lung lobe for histopathology (n = 5 per group). Created in BioRender. Britzke, T. (2026) https://BioRender.com/b602vx0.

(B-C) Body weight changes and survival of inoculated animals. Mean with standard deviation is shown.
(D-G) Viral RNA quantification by ORF1ab RT-qPCR in nasal washes **(D)** and organs at 2 dpi **(E)**, 5 dpi **(F)**, and 21 dpi **(G)**. Ordinary two-way ANOVA with Tukey´s multiple comparison test, with single pooled variance, based on log 10 values, was performed**.** *$p < 0.05$, ns = not significant**.** Mean with standard deviation is shown. Significant differences in nasal washes were observed between all groups, except between OTS-206 and OTS-228 at 3 and 5 dpi. The cut-off (3 gc/mL [$\log_{10}$]) is indicated by a dashed line.
(H) SARS-CoV-2-specific antibodies in serum of inoculated animals determined by RBD-ELISA and measurement of optical density (OD) at 450 nm. The ELISA cut-off (OD = 0.3) is indicated by a dashed line. Values above the cut-off were considered positive, indicating seroconversion.
(I-J) Body weight changes **(I)** and survival **(J)** of contact animals following co-housing with WT infected donors (1:1). Mean with standard deviation is shown.
(K-L) Viral RNA quantification in nasal washes **(K)** and organs **(L)** at 21 dpi of contact animals by ORF1ab RT-qPCR. The cut-off (3 gc/mL [log10]) is indicated by a dashed line.
(M) SARS-CoV-2-specific antibodies in serum of contact animals determined by RBD-ELISA and measurement of optical density (OD) at 450 nm. The ELISA cut-off (OD = 0.3) is indicated by a dashed line. Values above the cut off were considered positive, indicating seroconversion.

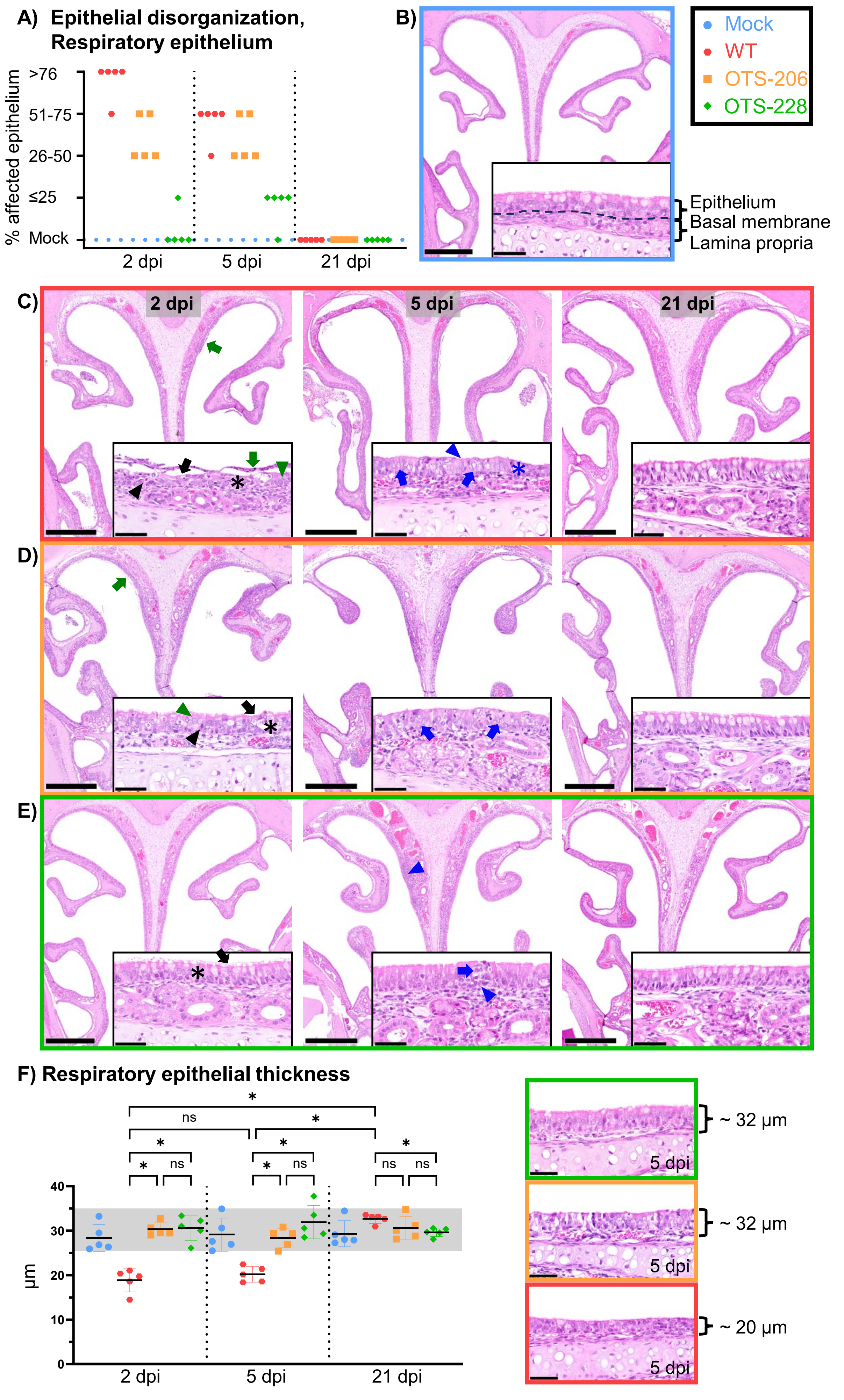

A) Epithelial disorganization, Respiratory epithelium
% affected epithelium
>76
51-75
26-50
≤25
Mock
2 dpi
5 dpi
21 dpi
B)
Mock
WT
OTS-206
OTS-228
Epithelium
Basal membrane
Lamina propria
C)
2 dpi
5 dpi
21 dpi
D)
E)
F) Respiratory epithelial thickness
µm
40
30
20
10
0
*
ns
2 dpi
5 dpi
21 dpi
~ 32 µm
~ 32 µm
~ 20 µm
5 dpi

Figure S2: Histopathology of the respiratory mucosa at 2, 5 and 21 dpi.
(A) Disorganization of the respiratory epithelium given in % showing gradual tissue damage for WT, OTS-206 and OTS-228 inoculation.
(B-E) Representative hematoxylin and eosin stained sections of nasal level 1 (overview, bar 500 µm; inlay bar 50 µm).
(B) Mock inoculated hamsters showed a well-structured respiratory mucosa.
(C) WT: At 2 dpi, extensive epithelial necrosis and loss (black asterisk), loss of the ciliary layer (black arrow), marked architectural disruption by infiltrating macrophages and granulocytes (black arrowhead). Remaining ciliated epithelial cells are rounded or flattened (green arrowhead); goblet cells lacked mucin granules, and cellular debris lined the luminal surface (green arrow). At 5 dpi, pronounced epithelial hyperplasia (blue arrow) with restoration of the ciliary layer (blue arrowhead) was observed, while epithelial organization remained markedly disordered (blue asterisk).
(D) OTS-206: At 2 and 5 dpi, numerous necrotic epithelial cells (black asterisk), and diffuse disruption by infiltrating macrophages and granulocytes (black arrow) were present; the epithelium remained continuous with largely preserved ciliated epithelial cells (green arrow), partial loss of the ciliary layer (black arrow), and minimal luminal debris (green arrow). Epithelial hyperplasia (blue arrow) was evident at 5 dpi.
(E) OTS-228: The epithelium is well preserved (black asterisk) with almost intact ciliary layer (black arrow), only single-cell necrosis (blue arrow) and mild epithelial disruption due to immune cell infiltration (blue arrowhead) at 5 dpi.
Complete epithelial recovery present at 21 dpi in all animals.
(F) Epithelial thickness significantly differed at 2 and 5 dpi: WT showed reduced thickness, whereas OTS-206 and OTS-228 maintained preserved thickness (bar 50 µm).
Ordinary one-way ANOVA with Tukey's multiple comparison was performed. Multiple virus variant comparisons (WT vs. OTS-206 vs. OTS-228) were performed at each dpi separately (2 or 5 or 21 dpi) and multiple time-point comparisons (2 vs. 5 vs. 21 dpi) were performed for each virus variant separately (WT or OTS-206 or OTS-228). *$p < 0.05$, ns = not significant. Mean with standard deviation is shown. Gray bar marks physiological limits. n=5 hamsters per group; 2, 5 and 21 dpi; nasal level 1-3 with in total 7 regions of interest were analyzed for the evaluation of the disorganization. The epithelial thickness was measured at the nasal septum at level 1.

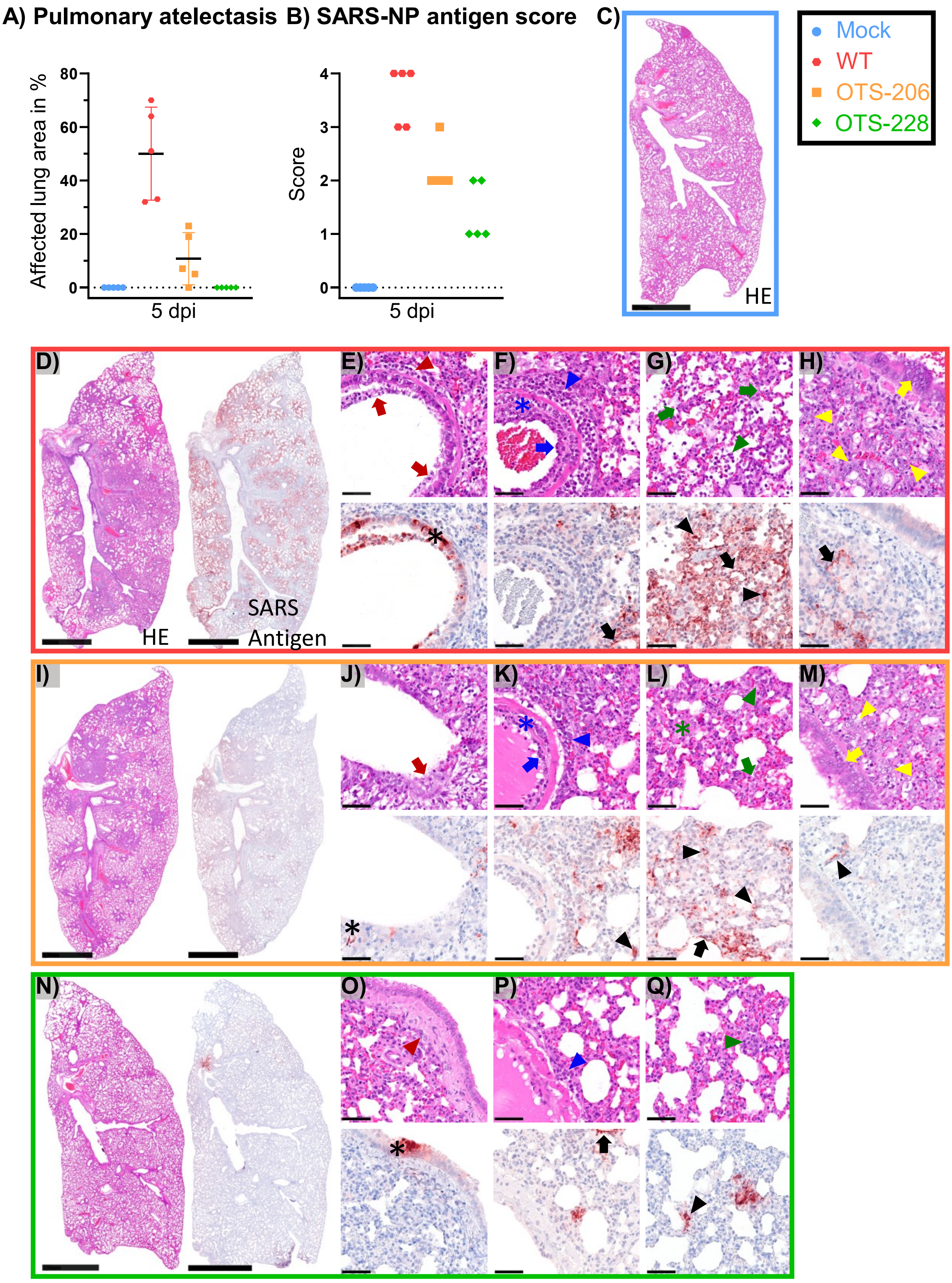

A) Pulmonary atelectasis
B) SARS-NP antigen score
C)
Affected lung area in %
80
60
40
20
0
5 dpi
Score
4
3
2
1
0
5 dpi
HE
Mock
WT
OTS-206
OTS-228
D)
E)
F)
G)
H)
HE
SARS
Antigen
I)
J)
K)
L)
M)
N)
O)
P)
Q)

Figure S3: Lung histopathology at 5 dpi.

(A) Pneumonia-associated pulmonary atelectasis (% affected area): WT induced moderate to severe atelectasis (33–70%), OTS-206 mild atelectasis (0–23%), and OTS-228 no atelectasis.

(B) SARS-NP labeling (semi-quantitative) showed coalescing (score 3) to diffuse (score 4) widespread antigen distribution after WT, predominantly multifocal clusters (score 2) of numerous positive cells after OTS-206, and only few cells in focal (score 1) or multifocal distribution after OTS-228.

(C-Q) Representative HE-stained whole lung sections (bar 2.5 mm), detail images (bar 50 µm), and corresponding SARS-NP IHC illustrate pathology and antigen distribution after mock **(C)**, WT **(D-H)**, OTS-206 **(I-M)**, and OTS-228 **(N-Q)** inoculation.

(D-H) WT: Moderate atelectasis with typical SARS-CoV-2 associated lesions, including **(E)** necrotizing bronchitis (red arrow) with marked peribronchial infiltrates (red arrowhead), **(F)** vasculitis (blue asterisk) with intravascular immune cell aggregation (blue arrow) and pronounced perivascular infiltrates (blue arrowhead), **(G)** extensive alveolar epithelial necrosis (green arrow), edema, and inflammatory infiltration predominantly by macrophages and granulocytes (green arrowhead), and **(H)** hyperplasia and hypertrophy of bronchial epithelial cells (yellow arrow) and type II pneumocytes (yellow arrowhead).

(I-M) OTS-206: Similar lesion types at lower severity, including **(J)** necrotizing bronchitis with peribronchial infiltrates, **(K)** vasculitis with immune cell aggregation and perivascular infiltrates, **(L)** predominantly interstitial macrophage infiltration (green arrowhead), mild alveolar infiltrates (green asterisk), and single-cell necrosis (green arrow), as well as **(M)** epithelial and pneumocyte hyperplasia/hypertrophy.

(N-Q) OTS-228: No atelectasis, necrotizing bronchitis, or vascular lesions; mild **(O)** peribronchial (red arrowhead) and **(P)** perivascular infiltrates (blue arrowhead) and **(Q)** slight interstitial expansion by macrophage-dominated inflammation (green arrowhead).

Viral antigen was detected in bronchial epithelial cells (black asterisk) and type I (black arrow) and II pneumocytes (black arrowhead) in all groups, correlating with lesions and inflammatory infiltrates.

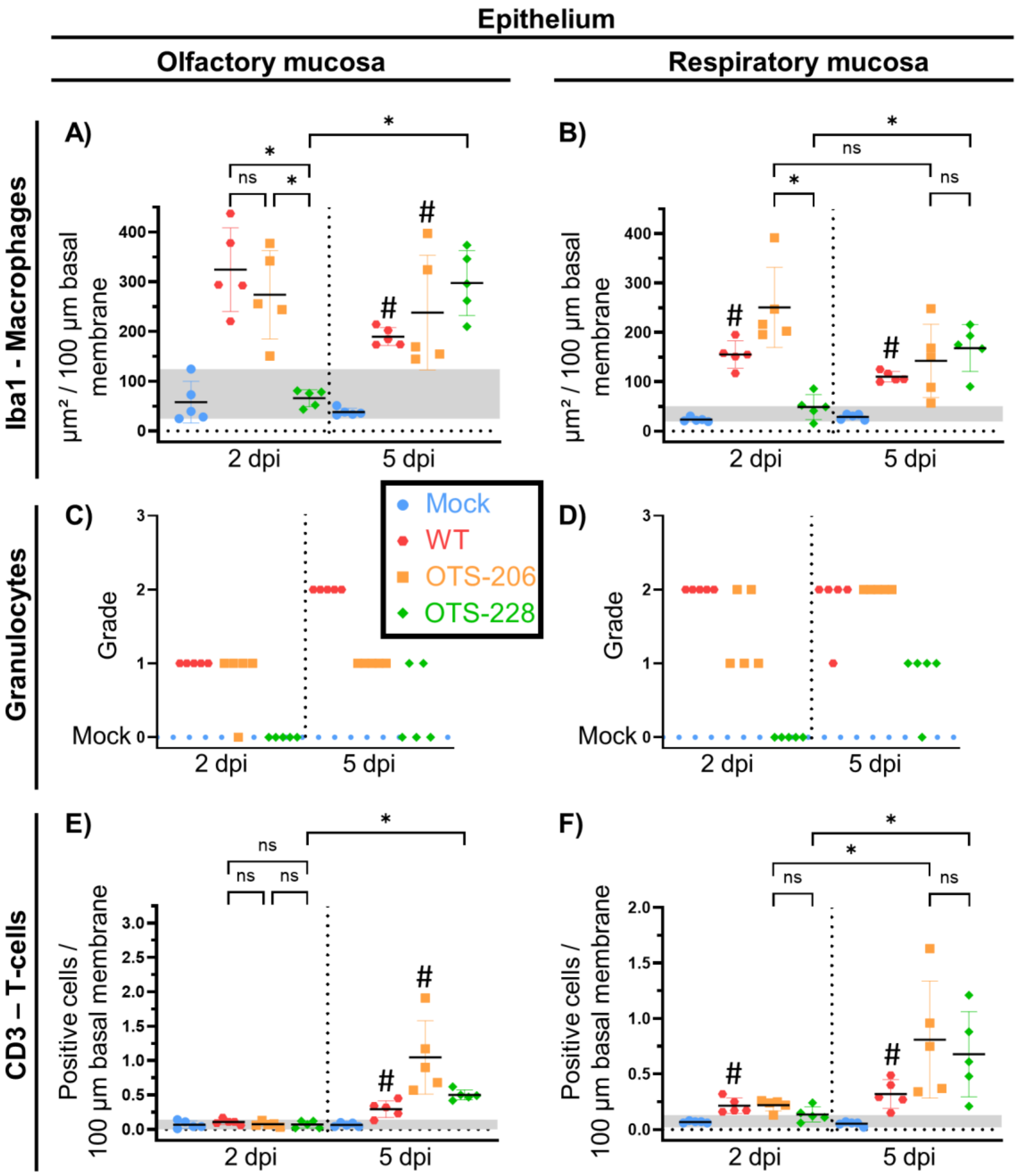

Figure S4: Immune cell infiltration in the epithelium of the olfactory and respiratory mucosa 2 and 5 days post WT, OTS-206 or OTS-228 inoculation.

(A-B) Iba1 labeling with digital image analysis showed macrophage infiltration in the olfactory epithelium **(A)** and respiratory epithelium **(B)**. At 2 dpi, macrophage areas were significantly increased for WT (olfactory epithelium only) and OTS-206 compared to OTS-228. At 5 dpi, OTS-228 showed a delayed but significant increase, reaching levels comparable to WT and OTS-206. For multiple group comparison at 2 dpi, one-way ANOVA with Tukey's multiple comparison (A) and Kruskal-Wallis test with Dunn's correction (B) were used; for pairwise comparisons, Welch's (A) or Mann-Whitney test (B) was applied.

(C-D) Semi-quantitative analysis revealed lower granulocyte infiltration in the olfactory epithelium **(C)** than in the respiratory epithelium **(D)** for all groups. WT showed the highest grades (moderate, grade 2), whereas OTS-228 showed the lowest grades (minimal, grade 1) at 5 dpi.

(E-F) CD3 labeling with digital image analysis showed T-cell infiltration in olfactory **(E)** and respiratory epithelium **(F)**, largely mirroring macrophage patterns, with a low but significant increase for OTS-228 at 5 dpi. For multiple group comparison at 2 dpi, Kruskal-Wallis test with Dunn's correction was used (E); for pairwise comparisons, Mann-Whitney test (E, F) was applied. *$p < 0.05$, ns not significant. # Not tested due to severe tissue damage and limited comparability. Mean with standard deviation is shown. Gray bar indicates physiological limits. n = 5 hamsters per group; 2 and 5 dpi; nasal levels 1-3 with in total 7 regions of interest were analyzed.

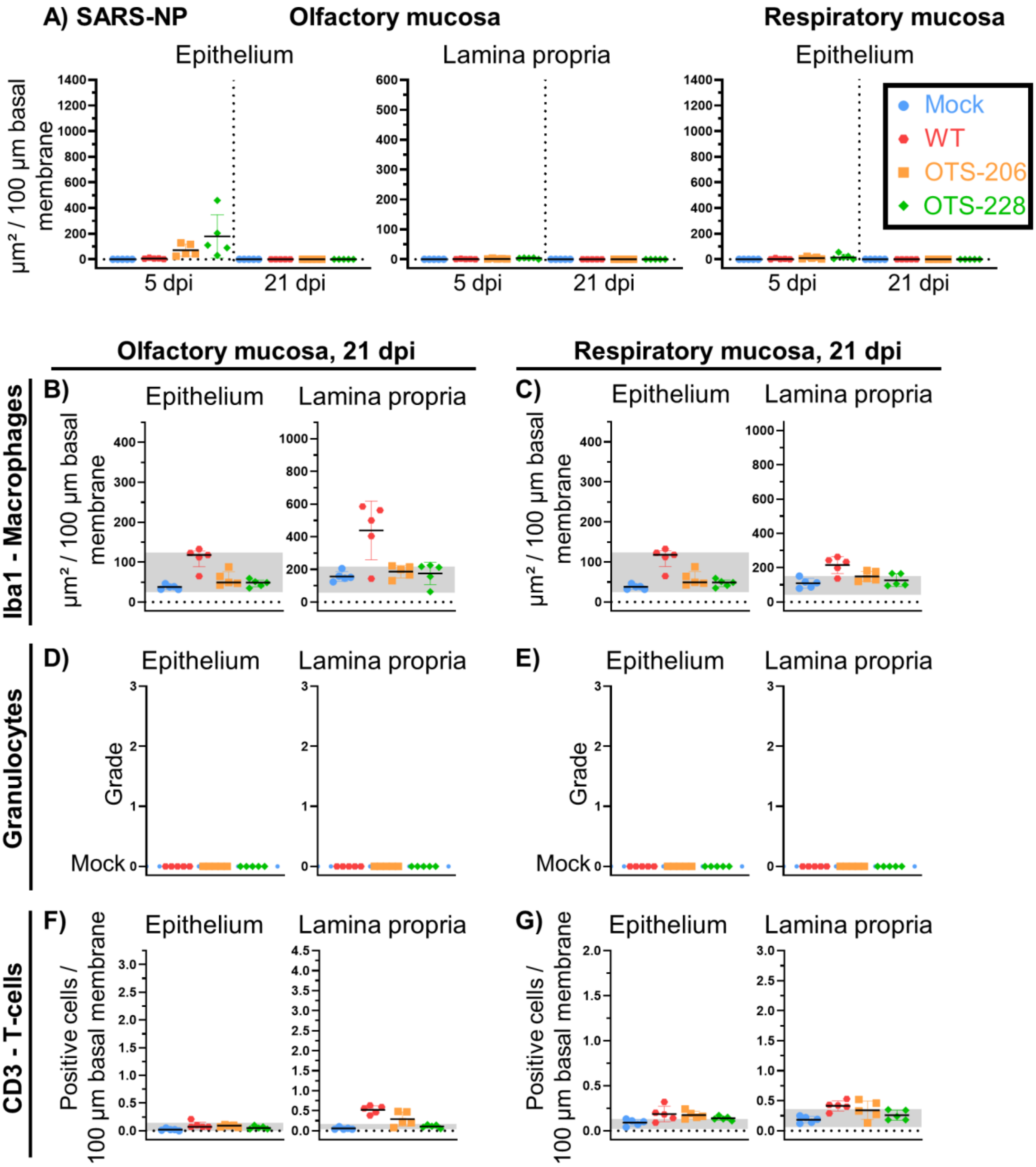

Figure S5: Viral antigen labeling and immune cell infiltration for the olfactory and respiratory mucosa 21 days post WT, OTS-206 or OTS-228 inoculation.
(A) SARS-NP labeling showed minimal to small antigen-positive areas at 5 dpi and absence of detectable antigen at 21 dpi in both olfactory and respiratory mucosa across all groups (WT, OTS-206, OTS-228).
(B-C) Iba1 labeling**, (D-E)** granulocyte evaluation, and **(F-G)** CD3 labeling showed values within or near physiological limits at 21 dpi. Residual macrophage and T-cell infiltrates were observed in some WT and OTS-206 inoculated animals. Mean with standard deviation is shown. Gray bar indicates physiological limits. n = 5 hamsters per group; 21 dpi; nasal levels 1-3 with in total 7 regions of interest were analyzed.

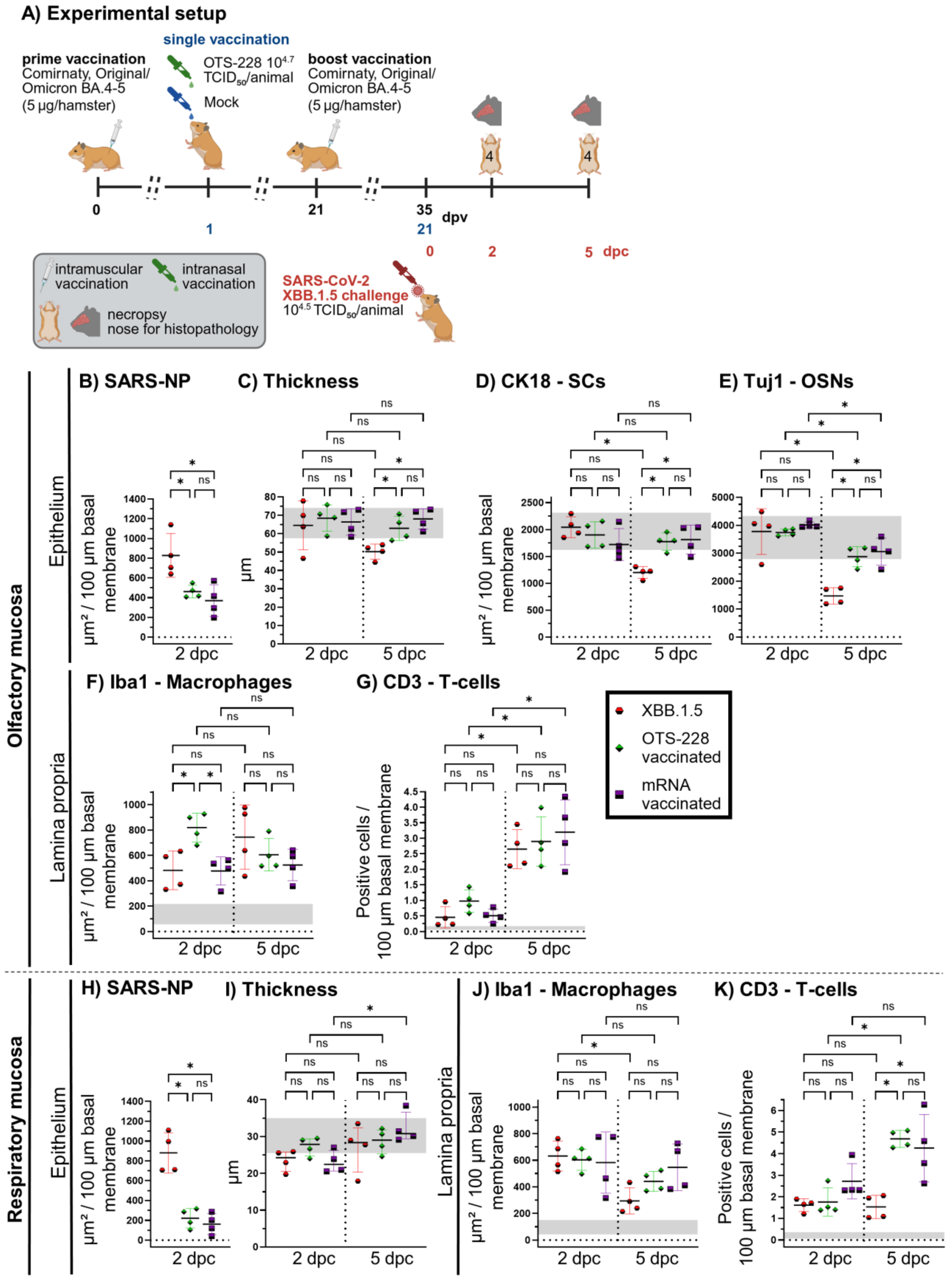

Figure S6: XBB.1.5 challenge of OTS-228 and mRNA vaccinated hamsters.

(A) Syrian hamsters (Mesocricetus auratus; female and male equally mixed; 5 weeks old) were vaccinated intramuscularly with a bivalent mRNA vaccine (Comirnaty, Original/Omicron BA.4-5; prime-boost), or intranasally with a single dose of OTS-228, or mock vaccinated, followed by challenge with SARS-CoV-2 XBB.1.5. Necropsies were performed at 2 and 5 days post challenge (dpc); heads were collected for histopathology (n = 4 per group). dpv: days post vaccination. Created in BioRender. Britzke, T. (2026) https://BioRender.com/akqz6b6.

(B-E) Olfactory epithelium: SARS-NP labeling showed significantly smaller antigen-positive areas in OTS-228 and mRNA vaccinated animals compared to unvaccinated controls **(B)**. Epithelial thickness **(C),** sustentacular cell (SC) area (CK18; **D**), and olfactory sensory neuron (OSN) area (Tuj1; **E**) remained within physiological limits in vaccinated animals.

(F-G) Olfactory mucosa, lamina propria: OTS-228 vaccinated animals showed significantly increased macrophage areas (Iba1) at 2 dpc compared to mRNA vaccinated and unvaccinated animals **(F).** All groups showed a comparable increase in T-cell numbers (CD3) at 5 dpc **(G).**

(H-I) Respiratory epithelium: SARS-NP labeling showed reduced antigen-positive areas in vaccinated animals compared to unvaccinated animals **(H),** while epithelial thickness did not differ between groups **(I).**

(J-K) Respiratory mucosa, lamina propria**:** Macrophage infiltration was comparable across groups **(J)**, but both OTS-228 and mRNA vaccination resulted in increased T-cell numbers at 5 dpc compared to unvaccinated animals **(K).**

For multiple group comparisons at 2 and 5 dpi, ordinary one-way ANOVA with Tukey's multiple comparison (B, C, D, E, F, G, H, I, K) or Kruskal-Wallis test followed by Dunn's correction (B, E, F, G, I, J, K) and for pairwise comparisons between time points, Welch's (C, D, E, F, G, I, K) or Mann-Whitney (E, F, G, I, J, K) test was performed. *$p < 0.05$, ns = not significant. Mean with standard deviation is shown. Gray bar marks physiological limits.

SARS-NP: SARS-CoV nucleocapsid protein; CK18: cytokeratin 18; Tuj1: neuron-specific class III β-tubulin; Iba1: ionized calcium-binding adapter molecule 1; CD3: cluster of differentiation 3. n = 4 hamsters per group; 2 and 5 dpc; nasal levels 1-3 with in total 7 regions of interest were analyzed. Epithelial thickness was measured at the nasal septum (level 3 for OE, level 1 for RE).

Table S1: Antibodies and reagents for immunohistochemistry

Antigen / Target	Species, Origin	Manufacturer, catalog #	Antibody Dilution	Antigen Retrieval	Secondary Reagents
SARS-NP / SARS-CoV*	Rabbit, polyclonal	Rockland (USA), 200-401-A50	1:3000	HIER, 10 mM citrate buffer, pH 6.0	Goat anti-rabbit IgG biotinylated, 1:200 (BA-1000); Vectastain Elite ABC Kit (both Vector Laboratories, USA)
Iba1 / macrophages	Rabbit, polyclonal	FUJIFILM Wako Pure Chemical Corporation (USA), 019-19741	1:500	HIER, 10 mM citrate buffer, pH 6.0	Goat anti-rabbit IgG biotinylated, 1:200; Vectastain Elite ABC Kit (BA-1000)
CD3 / T-cells	Rabbit, polyclonal	Agilent (USA), A0452	1:100	HIER, 10 mM citrate buffer, pH 6.0	Goat anti-rabbit IgG biotinylated, 1:200; Vectastain Elite ABC Kit (BA-1000)
PAX5 / B-cells	Rabbit, EPR3730(2)	Abcam (UK), ab109443	1:250	HIER, 10mM Tris/ 1mM EDTA buffer, pH 9.0	ImmPRESS goat anti-rabbit IgG Polymer Kit Peroxidase (MP-745, Vector Laboratories)
Tuj1 / olfactory sensory neurons	Mouse, 2G10	Thermo Fisher Scientific (USA), MA1-118	1:400	HIER, 10 mM citrate buffer, pH 6.0	Goat anti-mouse IgG biotinylated, 1:200 (BA-9200, Vector Laboratories); Vectastain Elite ABC Kit
CK18 / sustentacular cells	Mouse, CY-90	Merck KGaA (Germany), C-8541	1:800	HIER, 10mM Tris/ 1mM EDTA buffer, pH 9.0	Goat anti-mouse IgG biotinylated, 1:200 (BA-9200); Vectastain Elite ABC Kit
SOX2 / basal & sustentacular cells in OE	Rat, Btjce	Thermo Fisher Scientific, 14-9811-82	1:200	HIER, 10mM Tris/ 1mM EDTA buffer, pH 9.0	Goat anti-rat IgG mouse adsorbed affinity purified biotinylated, 1:50 (BA-9400 Vector Laboratories); Vectastain Elite ABC Kit

Abbreviations: SARS NP: SARS-CoV nucleocapsid protein; Iba1: ionized calcium-binding adapter molecule 1; CD3: cluster of differentiation 3; PAX5 paired box protein 5; Tuj1: neuron-specific class III beta-tubulin; CK18: cytokeratin peptide 18; SOX2: sex determining region Y box 2; OE: olfactory epithelium; HIER: heat-induced epitope retrieval; ABC: avidin-biotin-peroxidase complex solution; Tris/EDTA: tris(hydroxymethyl)aminomethane/ethylenediaminetetraacetic acid.
*SARS-NP with 90.5% sequence homology between SARS-CoV NP (NPCoVNC_004718) and SARS-CoV-2 NP Wuhan (NPnCoVNC_045512).

Table S2: Antibodies, buffer and reagents for immunofluorescence

Antigen / Target	Species, Origin	Manufacturer, catalog #	Antibody Dilution	Antigen Retrieval	Secondary Reagents
SARS-NP / SARS-CoV*	Rabbit, polyclonal	Rockland (USA), 200-401-A50	1:3000	HIER, 10mM Tris/ 1mM EDTA buffer, pH 9.0	Goat anti-rabbit Alexa Fluor 568, 1:500 (A11011, Thermo Fisher Scientific); Hoechst, 1:20.000 (94403, Merck KGaA)
CK18 / sustentacular cells	Mouse, Clone CY-90	Merck KGaA (Germany), C-8541	1:800		Goat anti-mouse Alexa Fluor 488, 1:500 (A11001, Thermo Fisher Scientific); Hoechst, 1:20.000
SARS-NP / SARS-CoV	Rabbit, polyclonal	Rockland, 200-401-A50	1:3000	HIER, 10 mM citrate buffer, pH 6.0	Goat anti-rabbit Alexa Fluor 568, 1:500 (A11011); Hoechst, 1:20.000
Tuj1 Tuj1 / olfactory sensory neurons	Mouse, 2G10	Thermo Fisher Scientific (USA), MA1-118	1:400		Goat anti-mouse Alexa Fluor 488, 1:500 (A11001); Hoechst, 1:20.000

**Permeabilization buffer**	PBS with 0.2% gelatin and 0.25% alkylphenyl polyethylene glycol
**Blocking buffer**	Permeabilization buffer with 5% bovine serum albumin
**Dilution buffer**	1% blocking buffer (diluted in PBS)
**Washing buffer**	10 mM $CuSO_4$ and 50 mM NH_4Cl in distilled water

Abbreviations: SARS N: SARS-CoV nucleocapsid protein; CK18: cytokeratin peptide 18; Tuj1: neuron-specific class III beta-tubulin; Mx1: Myxovirus resistance protein 1; HIER: heat-induced epitope retrieval; Tris/EDTA: tris(hydroxymethyl)aminomethane/ethylenediaminetetraacetic acid; PBS: phosphate buffered saline; DAPI: Diamidin-2-phenylindol.
*SARS-NP with 90.5% sequence homology between SARS-CoV NP (NPCoVNC_004718) and SARS-CoV-2 NP Wuhan (NPnCoVNC_045512).

Table S3: Probes (ACD Bio-Techne GmbH, Germany) and antibodies for RNA in-situ-hybridization

Probe, catalog #	Target gene	Opal fluorophore, catalog # (1:500)	Antigen / Target	Species Origin	Primary Antibody	Secondary Reagents
Mau-Ifnb1-C1, 1163061-C1	Interferon-beta 1 (IFN-β1)	Opal 690 Reagent, OP-001006				
Mau-Rigi-C3, 1848631-C3	Retinoic acid inducible gene I (RIG-I)	Opal 620 Reagent, OP-001004	SARS-NP / SARS-CoV*	Rabbit, polyclonal	1:3000 (200-401-A50, Rockland, USA)	Goat anti-rabbit IgG (H+L), peroxidase conjugated, 1:200 (PI-1000-1, Vector Laboratories, USA); Opal TSA-DIG Reagent, 1:250 (OP-001007); Opal 780 Reagent, 1:62.5 (OP-001008); DAPI
Mau-Mx1-C4, 1848641-C4	Myxovirus resistance protein 1 (Mx1)	Opal 570 Reagent, OP-001003				
Mau-Tnf-C1, 1062341-C1	Tumor necrosis factor-alpha (TNF-α)	Opal 570 Reagent, OP-001003	SARS-NP /SARS-CoV*	Rabbit, polyclonal	1:3000 (200-401-A50)	Goat anti-rabbit IgG (H+L), peroxidase conjugated, 1:200 (PI-1000-1); Opal TSA-DIG Reagent, 1:250 (OP-001007); Opal 780 Reagent, 1:62.5 (OP-001008); DAPI
Mau-Tnf-C1 1062341-C1	Tumor necrosis factor-alpha (TNF-α)	Opal 690 Reagent, OP-001006				
Mau-Il1b-C2 1062331-C2	Interleukin-1-beta (IL-1β)	Opal 620 Reagent, OP-001004	Iba1 / macrophages	Rabbit, polyclonal	1:500 (019-19741, FUJIFILM Wako Pure Chemical Corporation, USA), 1:500	Goat anti-rabbit IgG (H+L), peroxidase conjugated, 1:200 (PI-1000-1); Opal 520 Reagent, 1:500 (OP-001001); DAPI
Mau-Tgfb1-C4, 1848621-C4	Transforming growth factor β (TGF-β)	Opal 570 Reagent, OP-001003				

Mau-Mx1-C4, 1848641-C4	Myxovirus resistance protein 1 (Mx1)	Opal 690 Reagent, OP-001006	SARS-N / SARS-CoV	Mouse, 4F3C4	1:100 (In house, Labor Reiche, Friedrich-Loeffler-Institut)	Goat anti-mouse Alexa Fluor 488, 1:500 (A11001); DAPI, 1:20.000
			Mx1	Rabbit	1:100 (Cell Signaling Technology, USA)	Goat anti-rabbit Alexa Fluor 568, 1:500 (A11011); DAPI, 1:20.000
Positive control probes	Mau-Polr2a-C1, 890871-C1	RNA polymerase II subunit A				
	Mau-Ppib-C2, 890851-C2	Peptidylprolyl isomerase B				
	Mau-Ubc-C3, 890861-C3	Ubiquitin C				
	Mau-Hprt1-C4, 1848661-	Hypoxanthine-guanine phosphoribosyltransferase 1				
4-plex negative control probe	321831					

Abbreviations: SARS-NP / SARS-N: SARS-CoV nucleocapsid protein; Iba1: ionized calcium-binding adapter molecule 1; DAPI: Diamidin-2-phenylindol.
*SARS-NP with 90.5% sequence homology between SARS-CoV NP (NPCoVNC_004718) and SARS-CoV-2 NP Wuhan (NPnCoVNC_045512).

Table S4: HALO image analysis parameters for SARS, Iba1, Tuj1 and CK18 using Area Quantification v2.4.9

Antigen	Category	Average antigen stain color (OD)	Antigen minimal OD	Average hematoxylin stain color (OD)	Hematoxylin minimal OD	Blur radius
SARS NP	1	0.219; 0.607; 0.621	0.3554	0.242; 0.296; 0.200	0.1054	0.84
SARS NP	2	0.052; 0.159; 0.186	0.1946	0.233; 0.228; 0.132	0.1388	0.96
SARS NP	3	0.350; 0.725; 0.734	0.5344	0.327; 0.443; 0.346	0.1388	0.6
Iba1	1	0.213; 0.555; 0.561	0.3	0.289; 0.303; 0.181	0.25	0.75
Iba1	2	0.056; 0.264; 0.293	0.17	0.289; 0.303; 0.181	0.25	0.75
Iba1	3	0.264; 0.636; 0.636	0.45	0.289; 0.303; 0.181	0.25	0.75
Tuj1	1	0.146; 0.467; 0.514	0.155	0.245; 0.248; 0.151	0.15	0.35
Tuj1	2	0.074; 0.338; 0.394	0.085	0.200; 0.233; 0.154	0.15	0.35
Tuj1	3	0.373; 0.763; 0.773	0.4	0.365; 0.369; 0.230	0.2	0.35
CK18	1	0.248; 0.600; 0.600	0.38	0.411; 0.407; 0.233	0.325	0.42
CK18	2	0.084; 0.279; 0.299	0.2	0.411; 0.407; 0.233	0.325	0.42
CK18	3	0.327; 0.699; 0.699	0.55	0.411; 0.407; 0.233	0.325	0.42

Abbreviations: SARS N: SARS-CoV nucleocapsid protein; Iba1: ionized calcium-binding adapter molecule 1; Tuj1: neuron-specific class III beta-tubulin; CK18: cytokeratin peptide 18; OD: optical density

Protocol for setting the parameters of the Area Quantification v2.4.9 module:

The analysis magnification was set to 1x, and the real-time tuning window was activated to visualize how HALO applied parameter adjustments. The average pixel optical density (OD; stain color) in the RGB color spectrum was determined for both chromogen and hematoxylin signals, by selecting an area predominantly stained with chromogen or hematoxylin-stained pixels. Color deconvolution was used to verify whether HALO could effectively distinguish the two stains. If differentiation was insufficient, OD was recalibrated. Subsequently, the threshold for minimum OD was defined for both stains, establishing the sensitivity at which the software classified pixels as positive. Pixels below the threshold were considered negative, while those above were deemed positive. Finally, the blur radius was adjusted to smooth positive pixels and exclude very small positive areas. Optimization of OD thresholds and blur radius settings was performed in the real-time tuning window using the markup image.

Table S5: HALO image analysis parameters for CD3 using Multiplex IHC v3.4.9 (optical densities and thresholds)

			Nucleus Positive Threshold; Completeness Threshold		Cytoplasm Positive Threshold; Completeness Threshold		Membrane Positive Threshold; Completeness Threshold	
**Category**	**Average antigen stain color (OD)**	**Average hematoxylin stain color (OD)**	**Antigen**	**Hematoxylin**	**Antigen**	**Hematoxylin**	**Antigen**	**Hematoxylin**
**1**	0.373; 0.643; 0.607	0.357; 0.350; 0.184	0.21; 60%	0.125; 60%	0.18; 60%	-	0.2; 60%	-
**2**	0.123; 0.260; 0.270	0.255; 0.233; 0.142	0.07; 40%	0.1; 60%	0.07; 40%	-	0.07; 40%	-
**3**	0.342; 0.716; 0.699	0.407; 0.493; 0.327	0.225; 65%	0.125; 60%	0.225; 65%	-	0.225; 65%	-

Abbreviations: CD3: cluster of differentiation 3; OD: optical density

Table S6: HALO image analysis parameters for CD3 using Multiplex IHC v3.4.9 (nuclear and membrane parameters)

Category	Nuclear Detection Weight		Minimum Nuclear OD	Nuclear Size in µm² (min;max)	Minimum Nuclear Roundness	Nuclear Segmentation Aggressiveness	Maximum Cytoplasm Radius	Membrane Segmentation Aggressiveness	Cell Size in µm² (min;max)
	Antigen	Hematoxylin							
**1**	0.25	1	0.08	4;80	0	0.6	1.35	0.2	4;80
**2**	0.3	1	0.065	4;80	0	0.6	0.35	0.2	4;80
**3**	0.25	1	0.085	4;80	0	0.6	1.35	0.2	4;80

Abbreviations: CD3: cluster of differentiation 3; OD: optical density

Protocol for setting the parameters of the Multiplex IHC v3.4.9:

The magnification was set to 1x, the real-time tuning window was activated to enable live adjustment and verification of settings using color deconvolution and markup image visualization and the average pixel OD for both the chromogen and hematoxylin signals were determined. Subsequently, parameters for nuclear detection were configured. The nuclear segmentation type was set to AI Custom, and the classifier Nuclei Seg (Plugin) – BF v1.0.0 was selected. Nuclear detection weights were assigned to chromogen and hematoxylin signals to determine their impact on the identification of positive nuclei. A signal weight of 1 indicated maximum impact, whereas a weight of 0 excluded the signal from contributing to detection. The minimum nuclear OD threshold was established to determine the nucleus recognition. A stronger background required a higher threshold to avoid false positives, while weaker signals necessitated a lower threshold to ensure detection of faintly stained nuclei. To eliminate artifacts such as debris, the minimum nuclear roundness parameter was adjusted. Additionally, nuclear segmentation aggressiveness was adjusted to improve separation of clustered nuclei. The minimum and maximum nuclear area (in µm²) were also defined. Following nuclear detection, membrane and cytoplasm detection settings were configured. AI custom was selected for membrane segmentation type, with Membrane Seg (HALO AI) – BF v1.0.0 as classifier. The maximum cytoplasmic radius was specified to define positive signals attributed to a particular nucleus, thereby classifying the cell as positive. Membrane segmentation aggressiveness was adjusted to better distinguish adjacent cells. Minimum and maximum cell sizes (in µm²) were also defined. Finally, positive and completeness thresholds for both chromogen and hematoxylin signals were set for the nucleus, cytoplasm, and membrane compartments. The positive threshold defined the minimum OD at which pixels within a given cell compartment were considered positive. The completeness threshold specified the percentage of positive pixels required within a cell compartment, based on predefined nuclear and cellular size and segmentation parameters, for a cell to be counted as positive.

Supplemental notes 1: Attenuation and reduced viral replication of OTS vaccine candidates - clinical, virological and lung pathology data.

The majority of data when it comes to safety and efficacy evaluation of SARS-CoV-2 LAV candidates in the Syrian hamster, the most important natural SARS-CoV-2 preclinical model, are still clinical signs, viral genome loads in nasal washes and tissues and histopathology of the left lung lobe. As our main focus of investigation was on the primary replication site of SARS-CoV-2, the nasal cavity, we assed also these established readouts.

We compared intranasally OTS vaccinated hamsters (OTS-206 or OTS-228) with WT SARS-CoV-2 inoculated once and with a non-inoculated mock group (n=15 per group) using these classical readout. Of those donor animals were five per group sacrificed at 2 and 5 days post inoculation (dpi) each and the five remaining donor hamsters were further observed for body weight change and humoral immune respone until 21 dpi. At 1 dpi naïve contact animals co-housed in a 1:1 setup **(Figure S1A)**.

The WT infection led to significant weight loss, so that five animals met the human endpoint criteria at 5 and 6 dpi. In contrast, OTS-206 did not cause weight loss, premature death, but delayed and reduced the weight gain, whereas OTS-228 inoculated animals performed like the non-infected mock group **(Figure S1B-C)**. Viral genome shedding was detectable in nasal washings of all donors of all groups up to 12 dpi, peaking at 1 dpi, with significant differences between the groups, highest in WT, lowest in OTS-228 and OTS-206 in between **(Figure S1D)**. In tracheal and lung tissue at 2 and 5 dpi WT infected animals exhibited the highest viral loads, and OTS-228 the lowest **(Figure S1E-F)**. All donor animals confirmed virus genome negative at 21 dpi, except minimal amounts for OTS-206 animals, very close to the cut-off **(Figure S1G)**. All donor animals, except the mock animals, developed a SARS-CoV-2 specific humoral immune response at 21 dpi or even at 5 dpi in the WT and OTS-206 group **(Figure S1H)**.

The naïve direct contact animals exposed to WT infected donor animals exhibited body weight loss with a lowest mean of 93.2 % at 7 dpi, but did not show mortality **(Figure S1I-J)**. Virus transmission was confirmed by viral genome detection in nasal washing samples of 3 and 7 dpi (2 and 6 days after first contact) **(Figure S1K)**. Tissue samples of naïve direct contact animals of 21 dpi (20 days after first contact) confirmed mostly negative, except one trachea sample and genome traces in one lung sample **(Figure S1L)**. Transmission was further confirmed by serology of OTS-228 contact animals **(Figure S1M)**.

Histopathology of the lungs at 5 dpi revealed, that WT infection caused moderate to severe pneumonia-induced atelectasis, affecting up to 70% of the alveoli, accompanied by acute SARS-CoV-2 characteristic

vascular lesions, necrotizing bronchitis, extensive alveolar epithelial cell necrosis, marked perivascular and peribronchial infiltrates, and pronounced alveolar edema with infiltration, mainly by macrophages and granulocytes **(Figure S3A, D-H)**. OTS-206 exhibited comparable lesions but at lower grade **(Figure S3A, I-M)**. In contrast, neither atelectasis nor SARS-CoV-2 associated vascular lesions or necrotizing bronchitis were identified in OTS-228. Findings were limited to slight expansion of the interstitium by immune cell infiltration (5/5), mild perivascular (4/5) and peribronchial inflammatory infiltrates (4/5), predominantly macrophages **(Figure S3A, N-Q)**. Viral antigen was detected in type I and II pneumocytes and bronchial epithelial cells in all inoculated groups. In association with lesions, antigen levels were highest in WT, lower in OTS-206, and minimal in OTS-228 **(Figure S3B)**.

Supplemental notes 2: Detailed description of pathomorphological findings and disorganization of the nasal mucosa.

Pathomorphological findings following WT inoculation included widespread necrosis of epithelial cells in the olfactory epithelium (OE) at 2 dpi. Multifocal detachment of the epithelium led to exposure of the basal membrane. By 5 dpi, there was almost complete loss of the SCs and OSNs, only multilayered basal cells remained in the majority of the epithelium, and necrosis and loss extended to the proprias' Bowman's glands. The nasal cavity was filled with large amounts of cellular debris, proteinaceous mucus, and many degenerated granulocytes and macrophages. By 21 dpi, numerous SCs and OSNs including the ciliary layer were present again. Multifocal clusters of epithelial cells at the basal membrane indicated Bowman's glands regeneration **(Figure 3A, C)**. In the respiratory epithelium (RE), there was marked loss of the ciliary layer, extensive necrosis and loss of epithelial cells, and numerous infiltrating granulocytes and macrophages. Remaining ciliated epithelial cells are rounded or flattened, goblet cells lost their mucin granules. The epithelial cell layer was partially detached from the basal membrane, and partially covered by large amounts of cellular debris, mucus, and degenerated immune cells. By 5 dpi, there was pronounced hyperplasia of epithelial cells, with restoration of the ciliary layer. The RE of all animals was fully recovered at 21 dpi **(Figure S2A, C)**.

OTS-206 led to overall the same pathomorphological findings as described for WT. Though, in minimally affected animals, the basal membrane remained largely covered by SCs and OSNs. At 5 dpi, there was only partial loss of SCs and OSNs. Few sharply demarcated foci, with complete loss of SCs and OSNs were observed, with irregular, multilayered basal cells. Bowman's glands were largely preserved with only single cell necrosis. By 21 dpi, there was almost fully recovered epithelium including ciliary layer **(Figure 3A, D)**. In the RE, OTS-206 inoculation led to many necrotic epithelial cells, diffuse disruption of the epithelium by infiltrating granulocytes and macrophages but continuous and mainly preserved ciliated epithelial cells. Luminal cellular debri was minimally present. Complete recovery of the RE was seen at 21 dpi **(Figure S2A, D)**.

In clear contrast, not before 5 dpi, OTS-228 induced oligofocal single cell necrosis and mild disruption of the epithelium by immune cell infiltrations, but highly preserved, continuous epithelial cell layer and minimal cellular debris in the lumen. Of note, the dorsal nasal meatus was most consistently and severily affected with well demarced, focal detachement of the epithelium in 3 out of 5 hamsters. At 21 dpi, the OE appeared fully intact **(Figure 3A, E)**. The RE remained also well preserved. The ciliary layer appeared almost intact, and only few necrotic epithelial cells and oligofocal immune cell infiltrations were observed **(Figure S2A, E)**.

Disorganization of the OE after WT inoculation was moderate (51–75%, n=3/5) to severe (>76%, 2/5) at 2 dpi, progressing to severe (5/5) at 5 dpi and remaining moderate to severe at 21 dpi **(Figure 3A)**. In the RE, disorganization ranged from moderate (1/5) to severe (4/5) at 2 dpi and from mild (26–50%, 1/5) to moderate (4/5) at 5 dpi, with full recovery by 21 dpi **(Figure S2A)**.

OTS-206 induced minimal (≤25%, 3/5) to moderate (2/5) OE disorganization at 2 dpi, progressing to moderate (2/5) to severe (3/5) at 5 dpi. By 21 dpi, only minimal (3/5) to mild (2/5) disorganization remained and the epithelium was largely restored **(Figure 3A)**. In the RE, OTS-206 caused mild (3/5) to moderate (2/5) disorganization at 2 and 5 dpi, with full recovery by 21 dpi **(Figure S2A)**.

In contrast, OTS-228 caused only minimal alterations. OE disorganization was first detected at 5 dpi, ranging from minimal (4/5) to mild (1/5), most consistently affecting the dorsal nasal meatus. By 21 dpi, the OE was largely intact with only oligofocal minimal irregularities **(Figure 3A)**. The RE remained well preserved, with only minimal disorganization at 2 (1/5) and 5 dpi (4/5) **(Figure S2A)**.
